# Dysregulation of human ClpP using small molecules with piperazine-based scaffold for diffuse intrinsic pontine glioma therapy validated by patient-derived tumor organoids

**DOI:** 10.21203/rs.3.rs-8230151/v1

**Published:** 2026-01-14

**Authors:** Morena Miciaccia, Domenico Armenise, Olga Maria Baldelli, Anselma Liturri, Mariachiara Mammone, Giovanni Graziano, Marialessandra Contino, Francesca Rizzo, Paola Loguercio Polosa, Francesco Bruni, Clémence Deligne, Laura Broutier, Gianfranco Cavallaro, Cosimo Gianluca Fortuna, Anna Tolomeo, Cadell Green, Alessandro Bonifazi, Emily Majaesic, Marim M. Barghash, Nok Pang Josh Lee, Walid A. Houry, Carmen Gratteri, Carmine Talarico, Silvana Filieri, Giuseppe Micalizzi, Danilo Donnarumma, Paola Dugo, Luigi Mondello, Anna Maria Sardanelli, Javad Nazarian, Savina Ferorelli, Maria Grazia Perrone, Antonio Scilimati

**Affiliations:** 1 Research Laboratory for Woman and Child Health, Department of Pharmacy - Pharmaceutical Sciences, University of Bari “Aldo Moro”, 70125 Bari, Italy;; 2 Department of Pharmacy - Pharmaceutical Sciences, University of Bari “Aldo Moro”, 70125 Bari, Italy;; 3 Department of Biosciences, Biotechnologies and Environment, University of Bari “Aldo Moro”, 70125 Bari, Italy;; 4 Childhood Cancer & Cell Death Team (C3 Team), Consortium South-ROCK, LabEx DEVweCAN, Institut Convergence Plascan, Centre Léon Bérard, Centre de Recherche en Cancérologie de Lyon (CRCL), Université Claude Bernard Lyon 1, INSERM 1052, CNRS 5286, 69008 Lyon, France;; 5 Laboratory of Molecular Modelling and Heterocyclic Compounds ModHet, Department of Chemical Sciences, University of Catania, 95125 Catania, Italy;; 6 ITELPHARMA, ITEL Telecomunicazioni S.R.L., 70037 Ruvo di Puglia, Italy;; 7 Department of Pharmacology and Toxicology, Center for Addiction Sciences and Therapeutics, University of Texas Medical Branch, Galveston, TX 77555, USA;; 8 Department of Chemistry, University of Toronto, Toronto, Ontario M5S 3H6, Canada;; 9 Department of Biochemistry, University of Toronto, Toronto, Ontario M5G 1M1, Canada;; 10 LIGHT S.c.a.r.l., 25123 Brescia, Italy;; 11 Dompé Farmaceutici SpA, EXSCALATE, I-80131 Napoli, Italy;; 12 Department of Translational Biomedicine and Neuroscience, University of Bari “Aldo Moro”, 70124 Bari, Italy;; 13 Messina Institute of Technology c/o Department of Chemical, Biological, Pharmaceutical and Environmental Sciences, former Veterinary School, University of Messina, 98168 Messina, Italy;; 14 Chromaleont s.r.l., c/o Department of Chemical, Biological, Pharmaceutical and Environmental Sciences, former Veterinary School, University of Messina, 98168 Messina, Italy;; 15 Children’s Research Center, University Children’s Hospital Zurich, Zurich, Switzerland.

**Keywords:** piperazine, diffuse intrinsic pontine glioma (DIPG), ONC201 structure-activity relationships, hClpP, patient-derived DIPG organoids

## Abstract

Diffuse intrinsic pontine glioma (DIPG) is an aggressive brainstem tumor affecting children, with median survival under 12 months and no curative treatments. Current therapeutic development centers on ONC201, an imipridone that exhibits clinical activity by selectively activating human caseinolytic protease P (*h*ClpP). X-ray analysis of the *h*ClpP:ONC201 complex offers insights into optimizing the chemical structure of ONC201. Based on this, we designed and synthesized simplified piperazine-based analogs, identifying the polyfunctional chemotype **26** (DA29) through the systematic screening of five new series featuring piperazine-, piperazine-1-one (exocyclic carbonyl), piperazine-1,4-dione (exocyclic carbonyls), piperazine-2-one (endocyclic carbonyl), and piperazine-2,5-dione (endocyclic carbonyls) core scaffolds. The X-ray structure of the *h*ClpP:**26** (DA29) complex demonstrated a direct binding to the enzyme’s hydrophobic pocket, present at the apical domain, consistent with its strong proteolytic activation. Permeability studies under dynamic flow conditions indicated that **26** (DA29) can cross cellular barriers modeling drug transport, suggesting potential brain tumor penetration. Mechanistic studies showed that **26** (DA29) activates *h*ClpP, displaces *h*ClpX from the *h*ClpXP complex, induces mitochondrial dysfunction and causes ROS accumulation, exerts cytotoxicity and alters lipid profile in patient-derived DIPG cells with validation extended to patient-derived DIPG organoids for personalized medicine. The compound does not interact with D2/D3 receptors as ONC201 does, with validation extended to patient-derived DIPG organoids for personalized medicine. Overall, these findings establish a new structural framework for *h*ClpP-targeted therapy, representing a valuable step toward developing effective treatments for DIPG.

## Introduction

Diffuse intrinsic pontine glioma (DIPG), a subtype of diffuse midline glioma (DMG), accounts for about 10% of all paediatric brain tumors, typically diagnosed in children aged 4–10 years^1^. It presents with cranial nerve palsy, ataxia, and pyramidal tract abnormalities, and has a median survival of approximately 12 months, with life expectancy beyond progression rarely exceeding 3 months. Its localization in the brainstem, essential for vital functions such as breathing and cardiac activity, together with its diffuse growth pattern, prevents surgical resection, making therapy a major challenge in paediatric oncology^2^. Radiotherapy remains the standard treatment, yet prognosis is dismal, with over 90% of patients dying within 18–24 months of diagnosis.

**Figure 1.**
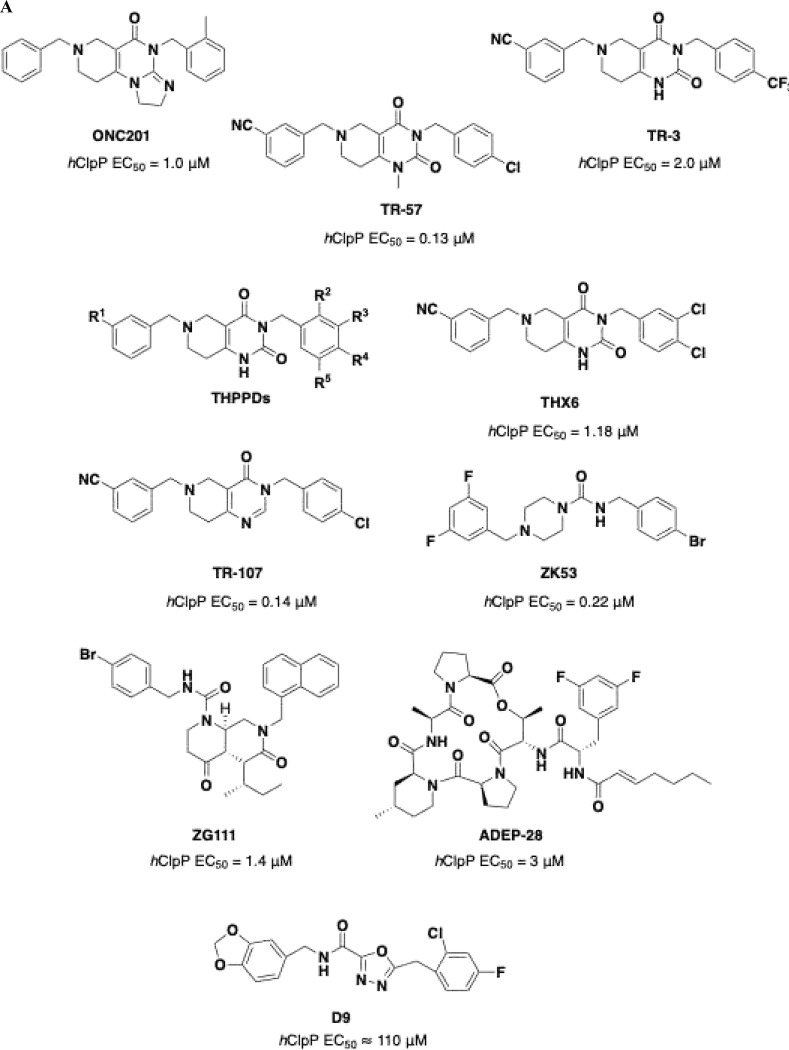

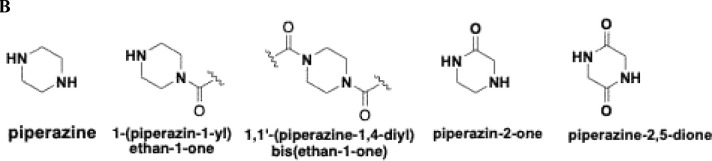
**A**) Chemical structures of **ONC201**, **TR-57**, **TR-3**, **THPPDs**, **THX6**, **TR-107**, **ZK53**, **ZG111**, **ADEP-28**, and **D9 and their EC**_**50**_ **(**half maximal effective concentration). **B**) Core scaffolds of piperazine, piperazine-1-one (exocyclic carbonyl), piperazine-1,4-dione (exocyclic carbonyls), piperazine-2-one (endocyclic carbonyl), and piperazine-2,5-dione (endocyclic carbonyls).

Extensive molecular analysis showed that the H3K27-alteration is present in approximately 80% of patients with DIPG^2–4^. Histones form chromatin and regulate gene expression through modifications such as methylation, phosphorylation, acetylation, and ubiquitination. Recurrent histone mutations, termed “oncohistones,” are frequently implicated in oncogenesis. Specifically, in DIPG, the replacement of lysine with methionine at position 27 of histone H3 (H3K27M) abolishes K27 methylation, causing epigenetic dysregulation in primitive neuronal stem and oligodendrocyte precursor cells. This H3K27M mutation blocks trimethylation by polycomb repressor complex 2, thereby driving oncogenic activity^1,5^. Growing scientific and clinical interest in DIPG led to the screening of approximately 200 molecules from the National Cancer Institute library, which identified **ONC201** (Fig. 1A). In August 2025, the FDA approved **ONC201**, under the name dordaviprone (Modeyso^™^), for recurrent H3K27M-mutant DMG, including DIPG^6^. Just two months earlier, the Italian Medicines Agency (AIFA) had approved the combination of nimotuzumab and vinorelbine for high-grade gliomas, after clinical trials showed a three-month improvement in overall survival^7^. **ONC201** inhibits cell growth and promotes apoptosis in various tumor types, with limited toxicity on normal cells. Initially described as an inducer of TRAIL-mediated apoptosis^8^, it was subsequently shown to act directly as a selective antagonist of the dopaminergic receptor DRD2 (*K*_*i*_ = 3 μM), overexpressed in several cancer types, including high-grade gliomas (h-GG) with H3K27M mutation, suppressing MAPK signaling, leading to tumor cell death in multiple preclinical models of h-GG^8^. However, DRD2 is not the only target of **ONC201**, as it also directly binds and activates human caseinolytic protease P (*h*ClpP), a serine protease located in the mitochondrial matrix. *h*ClpP together with the AAA+ unfoldase protein (ClpX) form the *h*ClpXP complex whose main function is to degrade damaged or misfolded proteins to prevent their accumulation, hence, preventing disruption of normal cellular function. Although the role of *h*ClpP in DIPG is not yet fully defined, its expression is upregulated in multiple solid tumors including lung, stomach, liver, thyroid, bladder, breast, ovary, prostate, testis, and brain, as well as in haematological malignancies^8–11^. Thus, modulators of *h*ClpP proteolytic activity may hold therapeutic potential. The activators of *h*ClpP interact with the enzyme allosteric pockets on the apical surface of the protein rigidifying the structure and favoring the extended active state^12^. Several activators of *h*ClpP are known, such as imipridones, acyldepsipeptides (**ADEPs**) and oxadiazonocarboxyamides (**D9**) (Fig. 1A) ^1,10^. The most extensively studied ClpP activators are imipridones, with **ONC201** as the prototype; later, *in silico* studies identified a tetrahydropyridopyrimidinone (**THPPD**)^13,14^ scaffold that yielded more potent *h*ClpP activators (e.g., **TR-57**, Fig. 1A).

Using **TR-57** and its derivative **TR-3** (Fig. 1A), we designed two new series of **THPPD**-based compounds (THX and THY) to investigate how benzyl substituents affect *h*ClpP activity, cytotoxicity in patient-derived DIPG cell lines, and cellular fatty acid profiles in the presence of the most promising analogs^2^.

In addition, we reported that the novel THPPD-related compound **THX6** alters the levels of proteins involved in key mitochondrial processes, including oxidative phosphorylation, organelle biogenesis, and mitophagy, suggesting that its anticancer activity may be linked to mitophagy inhibition^2^.

**SCHEME 1.**
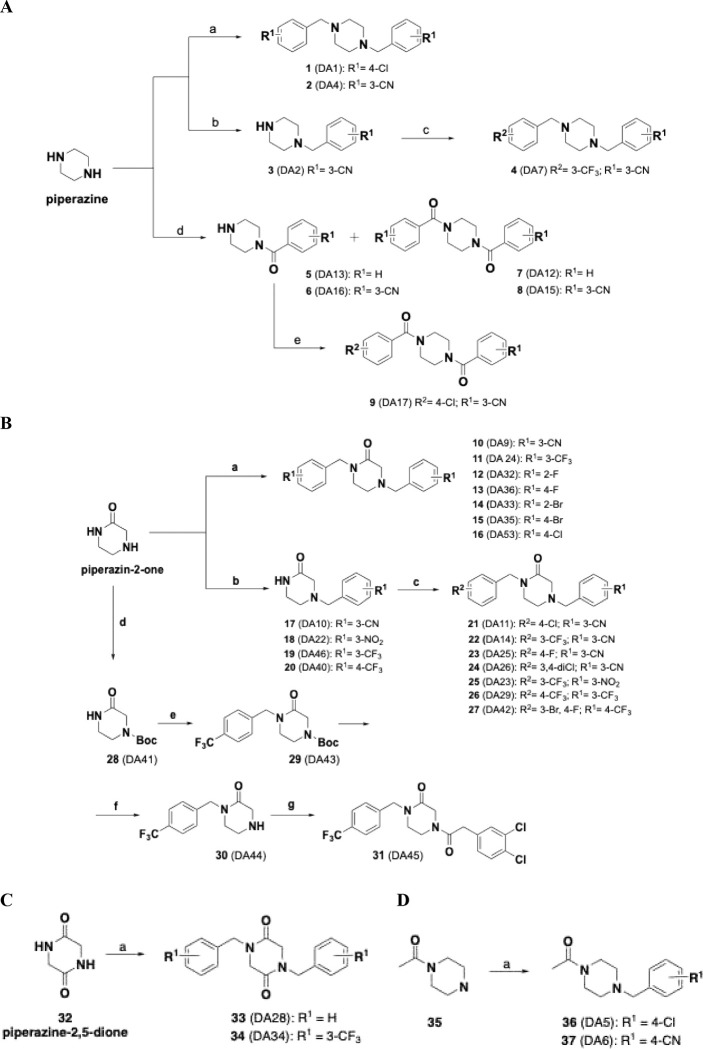
Reagents and conditions: **A**) a) aryl bromide, K_2_CO_3_ (2.5 eq), CH_3_CN, r.t., 2h; b) 4-chlorobenzyl bromide or 3-cyanobenzyl bromide, K_2_CO_3_ (1.3 eq), CH_3_CN, r.t., 2h; c) 3-trifluoromethylbenzyl bromide, K_2_CO_3_ (1.3 eq), CH_3_CN, r.t., 2h; d) benzoyl chloride or 3-cyanobenzoyl chloride (1 eq), absolute EtOH, 0 °C, r.t., overnight; e) 4-chlorobenzoyl chloride (1.5 eq), absolute EtOH, 0 °C, r.t., overnight. **B)** a) substituted-benzyl bromide, NaH, anhydrous DMF, 0 °C for 30 min, 50 °C, overnight; b) substituted-benzyl bromide, NaHCO3, anhydrous DMF, 100 °C, overnight; c) substituted-benzyl bromide, NaH, anhydrous DMF, 0 °C for 30 min, 50 °C overnight; d) di-*tert*-butyl decarbonate, CH_2_Cl_2_, 0 °C to r.t., 3h; e) substituted-benzyl bromide, anhydrous DMF, NaH, 0 °C, 30 min, 50 °C, overnight; f) CF_3_CO_2_H, CH_2_Cl_2_, r.t., 5h; g) 2-(3,4-dichlorophenyl)acetyl chloride, CH_2_Cl_2_, r.t. overnight. **C**) a) substituted-benzyl bromide, NaH, anhydrous DMF, 30 min, r.t., 16h; **D**) a) substituted-benzyl bromide, K_2_CO_3_, CH_3_CN, r.t., 2h.

As a further step beyond **THPPD** compounds, simplified analogs of **ONC201**, designed based on systematic point structural modification of core scaffolds consisting of piperazine, piperazine-1-one (exocyclic carbonyl), piperazine-1,4-dione (exocyclic carbonyls), piperazine-2-one (endocyclic carbonyl), and piperazine-2,5-dione (endocyclic carbonyls) (Fig. 1B), have been synthesized and characterized as novel *h*ClpP activators and are described herein (Scheme 1A–D, Table 1).

These novel piperazines resemble **ZK** compounds, such as **ZK53** (Fig. 1A), a potent and selective *h*ClpP activator (EC_50_ = 0.22 μM) studied *in vitro* and *in vivo* lung squamous carcinoma^11^. This rational scaffold modification strategy, obtained simplifying **ONC201** and **THPPD** through stepwise chemical modifications, allowed the identification of structural determinants enhancing *h*ClpP proteolytic activity and the development of polyfunctional chemotypes.

## Results and discussion

### Rational design of piperazines

The remarkable effectiveness of **THPPDs** as *h*ClpP activators prompted us to design a new scaffold capable of establishing stronger interactions with the allosteric site of the enzyme and further positively enhancing its proteolytic activity. Starting from the essential interactions established by **ONC201** and **THPPDs** with *h*ClpP, a targeted process of structural simplification was carried out to select essential structural determinants for optimal enzymatic binding.

In **ZK53, ONC201** and **THPPDs**, the core ring contains two nitrogen atoms and zero, one, or two endocyclic carbonyl groups. In addition, **ONC201** and **THHPDs** have the nitrogen in the 1,3-positions of an “unsaturated” piperidine, whereas **ZK53** bears nitrogen atoms in the 1,4-positions of a “saturated” piperazine with an exocyclic carbonyl. Since these combinations had already been explored, we designed a “saturated” ring bearing the two nitrogen atoms in 1,4-position, yielding simplified scaffolds based on piperazine, piperazine-1-one (exocyclic carbonyl), piperazine-1,4-dione (exocyclic carbonyls), piperazine-2-one (endocyclic carbonyl), and piperazine-2,5-dione (endocyclic carbonyls), incorporating endo- or exo-cyclic carbonyl(s) (Fig. 1B) but avoiding the pseudo-ureidic moiety of **ZK53** (Fig. 1A)^2^.

### Mapping critical enzyme regions for targeted drug development

The rational design of piperazine derivatives, with or without endo- or exocyclic carbonyl(s), was followed by their synthesis (Schemes 1A–D) and *in vitro* extensive evaluation. Piperazines lacking carbonyl groups did not activate *h*ClpP at concentrations up to 100 μM evaluated by a cell-free fluorimetric assay. Further modification of the piperazine core was therefore hypothesized to enhance interactions with the enzyme, based on the essential contacts established for **ONC201** and **ZK53** within the allosteric binding site of *h*ClpP.

**Figure 2.**
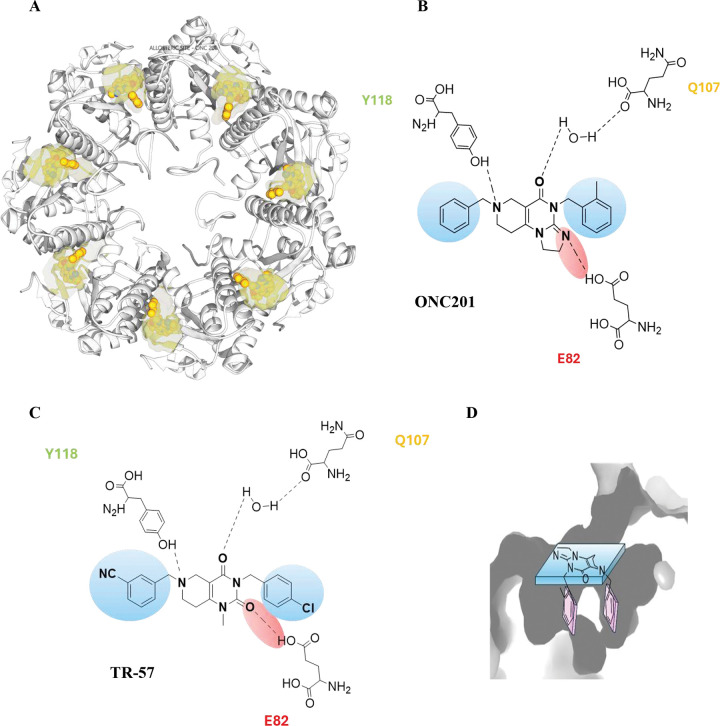
**A**) PDBID: 6DL7, complex with **ONC201** (orange space fill). The protein is shown in white cartoon while the identified allosteric binding sites, revealed by GENEOnet, are shown in yellow surface. The allosteric binding sites are those related to the co-crystallized **ONC201**. **B**) Main interactions of *h*ClpP with **ONC201** and **C**) **TR-57**. In **ONC201**, the carboxyl of E82 interacts with the imidazole nitrogen atom, whereas in **TR-57** the same residue interacts with a carbonyl. These specific interactions are highlighted in red and light blue. **D**) **ONC201** in the U-shaped conformation (pincer topology).

To evaluate which regions are involved in the binding with the enzyme, a binding site mapping was carried out using GENEOnet* software^15^ finding the known catalytic site, and the already reported allosteric binding sites, referring to the 3D structures related to the *h*ClpP:TR-57, *h*ClpP:ONC201 and *h*ClpP:ZK53 complexes (PDBID: 7UVN, 6DL7 and 8HGK). On all the three structures, the top-ranked binding sites are the regions comprehending the catalytic site and the binders **TR-57**, **ONC201** and **ZK53**, respectively. The druggability scores assigned are between 0.78 and 0.81, suggesting and confirming a high propensity of that region to bind a small molecule. Fig. 2A shows the binding site analysis made on 6DL7, selected given its higher resolution than others.

Analysis of the *h*ClpP:TR-57 complex (PDBID: 7UVN) revealed the essential structural features that enable **THPPDs** to act as *h*ClpP activators (Fig. 1). These compounds possess a central planar, electron-deficient heterocycle and two benzyl groups that establish hydrophobic π-π stacking interactions within the allosteric site. Their binding promotes ClpX dissociation, stabilization of the ClpP tetradecamer, and enlargement of the axial pore.

Furthermore, hydrogen-bonding interactions are critical for stabilization: the nitrogen atom of the tetrahydropyridopyrimidinone (**THPPD** and **TR-57** core) forms a hydrogen bond with the hydroxyl group of tyrosine (Y118), while the carboxyl engages in a water-mediated hydrogen bond with the terminal amide of glutamine (Q107). A further hydrogen bond involves the carboxyl of glutamic acid (E82), which interacts with the imidazole nitrogen atom of **ONC201** and related imipridones. In derivatives bearing the tetrahydropyridopyrimidinone core, such as **TR-57**, this same E82 carboxyl interacts instead with a second carboxyl group in the bicyclic scaffold (Fig. 2B).

The benzyl residues are located within two lateral hydrophobic pockets, containing L79, L104, and F105. Additionally, there is a series of aromatic amino acids, including Y118, Y138, and W146, which collectively determine the ligand positioning in a “U-like” conformation (pincer topology) (Fig. 2D).

Based on the finding that simpler compounds such as **ZK53**, compared to the imipridone **ONC201** and THPPDs, still act as *h*ClpP activators, we initiated a deeper structure-activity relationship study using piperazine-based scaffolds. A series of derivatives was synthesized and evaluated, featuring as central cores piperazine, piperazine-1-one (exocyclic carboxyl), piperazine-1,4-dione (exocyclic carboxyls), piperazine-2-one (endocyclic carboxyl), and piperazine-2,5-dione (endocyclic carboxyls) (Fig. 1B).

### Chemistry

The synthesis of piperazines (Scheme 1A) **1** (DA1) and **2** (DA4) was performed by reacting the commercially available piperazine with the corresponding 4-chlorobenzyl bromide or 3-cyanobenzyl bromide, respectively, in the presence of K_2_CO_3_ (2.5 equivalents); similarly, the monosubstituted piperazine **3** (DA2) was prepared using a smaller amount of K_2_CO_3_ (1.3 equivalents). Piperazine treated with the appropriate benzoyl chloride, in turn prepared from the corresponding benzoic acid and SOCl_2_, afforded the mono-benzoylpiperazines **5** (DA13) and **6** (DA16), and the 1,4-dibenzoylpiperazine **7** (DA12) and **8** (DA15). Finally, 3-(4-(4-chlorobenzoyl)piperazine-1-carbonyl)benzonitrile (**9**, DA17) was obtained by reacting 3-(piperazine-1-carbonyl)benzonitrile (**6**, DA16) with 1.5 equivalents of 4-chlorobenzoyl chloride.

The commercial 2-oxopiperazine (piperazine-2-one) was treated the appropriate benzyl bromide in dry DMF in the presence of NaH, to obtain the symmetric substituted oxopiperazines **10**-**16**. Monosubstituted derivatives **17**-**20** were obtained by reacting 2-oxopiperazine, in the presence of the weaker base NaHCO_3_, with the appropriate benzyl bromide. **17–20** treated with the corresponding substituted-benzyl bromide in dry DMF in the presence of NaH, allowed the synthesis of **21**-**27** (Scheme 1B). Compound **31** (DA45) was prepared starting from 2-oxopiperazine *N*-Boc protected **28** (DA41), in turn converted into **29** (DA43) by reacting with 4-trifluormethylbenzyl bromide, the later then converted into **30** (DA44) after deprotection of **29** (DA43) **30** (DA44) reacted with 2-(3,4-dichlorophenyl)acetyl chloride to afford **31** (DA45) (Scheme 1B).

**33** (DA28) and **34** (DA34) were prepared by reacting the piperazine-5,6-dione (**32**) with benzyl bromide and 3-cyanobenzyl bromide, respectively, in anhydrous conditions and in the presence of NaH (Scheme 1C). **36** (DA5) and **37** (DA6) were prepared in good yields by reacting the 1-(piperazin-1-yl)ethan-1-one (**35**) and 4-chlorobenzyl bromide or 4-cyanobenzyl bromide, respectively, in the presence of K2CO3 (Scheme 1D).

### Biology

#### Evaluation of human ClpP activation rate

**Figure 3.**
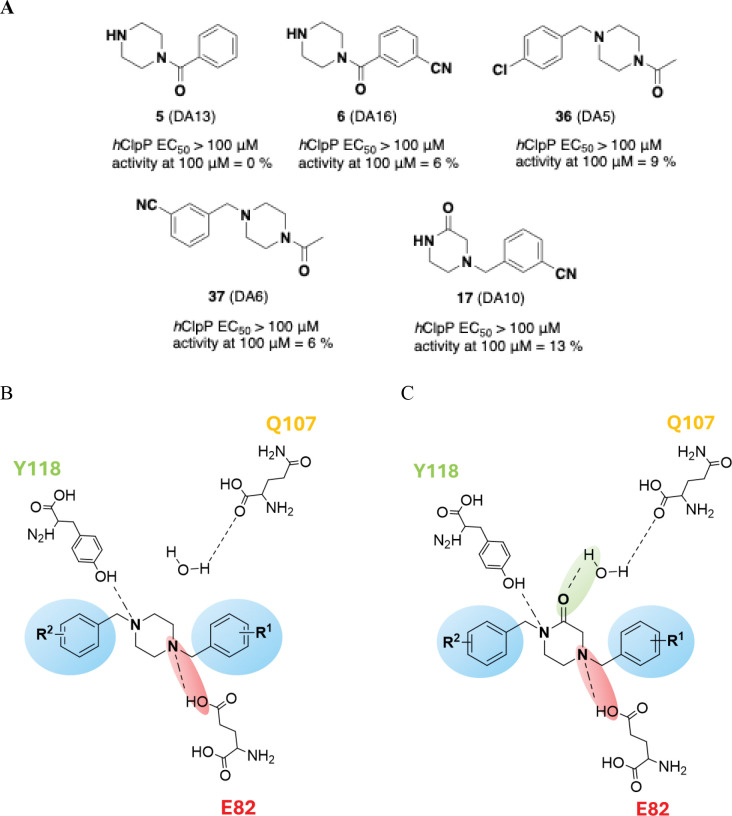
**A**) Some precursors of the target compounds bearing only one benzyl. **B).** On the left there are piperazine-core interactions, where the interaction with Q107 is lost; right, there are dibenzyl 2-oxopiperazine derivatives where the carboxyl interaction with Q107 is restored.

The new piperazines were designed to enable interactions with key *h*ClpP residues, aiming to reproduce the typical U-shaped conformation with two benzyls positioned in the hydrophobic pockets (Fig. 2D). As a preliminary step, precursors bearing only one benzyl were tested for their ability to enhance *h*ClpP proteolytic activity evaluated using a fluorimetric cell-free assay based on the cleavage of FITC-labeled casein substrate, with activity monitored as an increase in fluorescence intensity over time. Compounds **5** (DA13), **6** (DA16), **36** (DA5), and **37** (DA6), considered precursors of **ZK53** with an exocyclic carboxyl, and compound **17** (DA10), with an endocyclic carboxyl as in **THX6**^2^, were evaluated. All showed low potency as *h*ClpP activators, with EC_50_ values >100 μM and residual *h*ClpP activation in the range 0–13% (Fig. 3A).

In the series of piperazines lacking carboxyl groups, either endo- or exocyclic such as **1** (DA1), **2** (DA4), and **4** (DA7), only weak *h*ClpP activation was observed (EC_50_ >100 μM, 9–27% activation at 100 μM; Scheme 1A, Table 1). This limited activity is attributed to the absence of carboxyl-mediated interaction with residue Q107, as seen in imipridones (i.e. **ONC201**), **TRs**, and **THPPDs** (Fig. 1A, 2A and 2B). Nonetheless, residual activity may result from preserved interactions of the two piperazine nitrogen atoms with E82 and Y118, together with the contribution of benzyl substituents bearing electron-withdrawing groups (EWG) (mild EWG effect from chlorine, strong EWG effect from cyano group) (Fig. 3B).

In an attempt to strengthen the binding force with the enzyme, a carboxyl external to the piperazine core was introduced. Piperazines **7 (**DA12), **8 (**DA15) and **9 (**DA17) were obtained; EC_50_ > 100 μM and 9–11 % activation at 100 μM were found for all the three compounds. These results clearly showed that the carboxyl must be endo-cycle at least for these compounds, despite **ZK53** having an exocyclic carboxyl.

To further evaluate the role of carboxyl groups, new derivatives were designed with a 2-oxopiperazine core, where the carboxyl at position 2 interacts with residue Q107.

Unfortunately, this interaction seems not enough to sustain *h*ClpP proteolytic activity: compound **10** (DA9) showed EC_50_ > 100 μM likely due to the cyano substituent on the left benzyl group rendering the phenyl electron-poor and unable to establish strong π-π interactions. By contrast, compound **11** (DA24) achieved a significant increase in *h*ClpP-mediated hydrolysis, with 85% activation and an EC_50_ = 12 ± 5.15 μM. In this series, the proteolytic activity was strongly affected by the substituents on the aromatic moieties of the two benzyl groups, increasing with electron-withdrawing effects, particularly on the right-hand benzyl, which favored anchoring within the hydrophobic pockets (Fig. 3B). No halogen effect was observed for fluoro- and chloro-substituted compounds **12** (DA32), **13** (DA36), **14** (DA33) and **16** (DA53) having EC_50_ >100 μM. In contrast, the effect of bromine depended on its position: *ortho* substitution increased activity by 24% at 100 μM, while *para* substitution markedly enhanced activation to 91% at the same concentration [EC_50_ = 6.08 μM for **15** (DA35)]. Introducing the cyano or nitro group (very strong EWG) on the right benzyl ring yielded new activators [**21** (DA11), **22** (DA14), **23** (DA25), **24** (DA26) and **25** (DA23)], with EC_50_ values of 15–30 μM. The most potent compound, **26** (DA29), combined a 3-CF_3_ substituent on the right benzyl with a 4-CF_3_ on the left, achieving EC_50_ = 0.85 ± 0.14 μM. However, replacing the 3-CF_3_ with two halogens (3-Br, 4-F) abolished activity (EC_50_ >100 μM), even when incorporated into a piperazine-2-one [**27** (DA42)] or a piperazine-2-one bearing an additional exocyclic carboxyl [**31** (DA45)]. Similarly, derivatives with a second endocyclic carboxyl [**33** (DA28), **34** (DA34)] remained inactive (EC_50_ >100 μM).

The drug-stimulated proteolytic activity of *h*ClpP was evaluated using a fluorimetric assay based on the cleavage of FITC-labeled casein substrate, with activity monitored as an increase in fluorescence intensity over time. Selected compounds [**9** (DA17), **11** (DA24), **12** (DA32), **14** (DA33), **15** (DA35), **16** (DA53), **26** (DA29), **27** (DA42), **31** (DA45), **33** (DA28), and **34** (DA34)] displayed marked selectivity for *h*ClpP, showing negligible affinity for dopamine D2 and D3 receptors (Ki >10,000 nM, Table S1). By contrast, **ONC201** binds *h*D2R and *h*D3R with Ki values of 14.8 ± 1.40 μM and 4.09 ± 0.69 μM, respectively, consistent with its dual mechanism of *h*ClpP activation and dopamine receptor antagonism^2^. These results confirm that the tested compounds selectively target *h*ClpP with minimal off-target activity on D2-like receptors (Table S1). In the crystal structure (PDBID: 8HGK), FLAP and Autodock Vina analyses showed that **26** (DA29) binds in a mode comparable to **ZK53**, engaging, Y118, V145 and W146 with enhanced interactions and a more favorable binding energy (−10.3 vs −9.3 kcal/mol, Fig. S1).

### *h*ClpP:26 (DA29) complex structural analysis by X-ray

**Figure 4.**
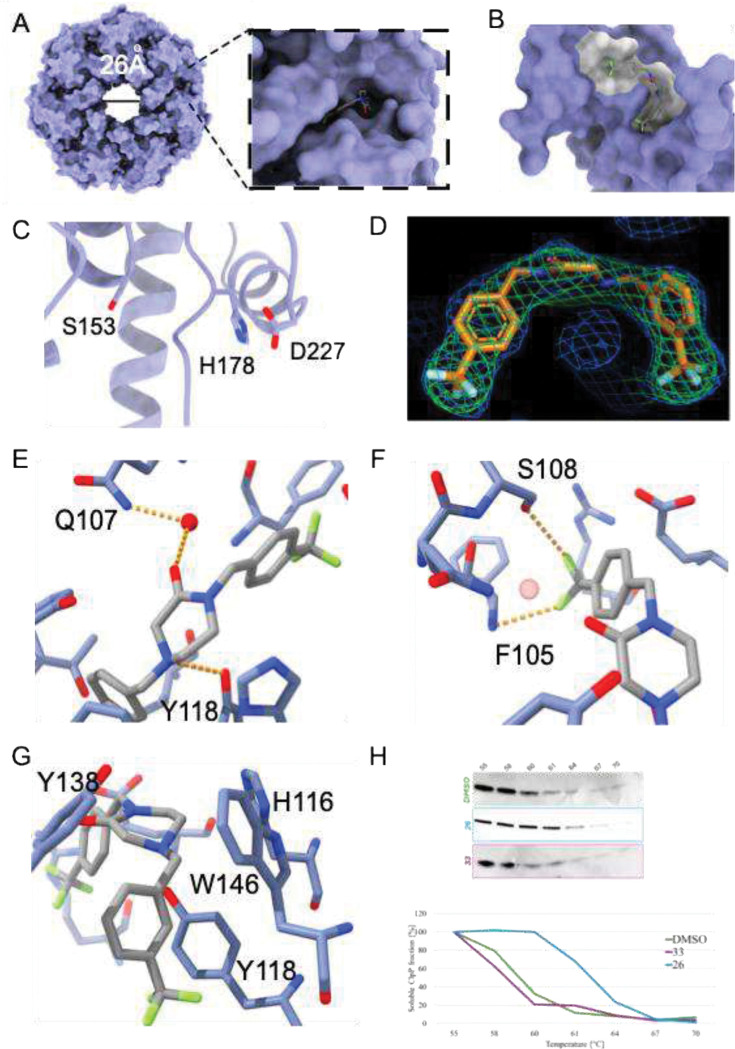
26 (DA29) binding *h*ClpP. (**A**) Top view of *h*ClpP showing enlarged pore diameter and **26** (DA29) bound in all hydrophobic pockets. Inset shows an enlarged view of **26** (DA29) bound to the pocket. (**B**) **26** (DA29) adopts pincer (U-shaped) topology to dock into both cavities. (**C**) Geometry of the catalytic triad in **26** (DA29)-bound *h*ClpP**.** S153, H178, D227. (**D**) Electron density at the hydrophobic pocket; 2Fo-Fc maps are colored blue and contoured at 1.0σ, while composite omit maps are colored green and contoured at 1.8σ **E**, **F**, **G).** (**E**) Shown are interactions between **26** (DA29) (grey) and *h*ClpP (purple) highlighting the hydrogen bond interactions (yellow) with the piperazine-1-one ring. (**F)** Hydrogen bond interactions (yellow) with the para-substituted arm. (**G**) Hydrophobic and π-π interactions between the meta-substituted arm. (**H**) Cellular thermal shift analysis of the thermal stability of *h*ClpP in Caco-2 cells treated with **26** (DA29), **33** (DA28) and DMSO at 20 μM for 1h at 55 °C.

To validate the binding interactions between **26** (DA29) and *h*ClpP, the X-ray structure of the *h*ClpP:**26** (DA29) complex was determined at 2.78 Å. Crystallographic data collection and refinement statistics are shown in Table S2. *h*ClpP crystallized as a tetradecamer with fourteen **26** (DA29) molecules bound in the hydrophobic pockets at subunit interfaces (Fig. 4A, B). The complex adopts an open pore compact conformation consistent with previously reported *h*ClpP structures bound with activators (e.g. **ONC201**, **ZK53, TR-57**)^11,16–17^. In the apo state, the flexible *N*-terminal regions cover the axial pore of *h*ClpP restricting substrate access to the catalytic chamber^18,19^. Upon agonist binding, these loops undergo ordering into a collar of β-hairpins that results in pore widening^18,19^. In the **26** (DA29)-bound structure, crystal packing prevented full resolution of the *N*-terminal loops however partially resolved chains indicate a clear ordering of this region. The axial pore radius is widened to 26 Å compared to the 13 Å radius of the apo structure (PDBID: 1TG6)^16^ (Fig. 4A, B). The αE helices in the **26** (DA29)-bound structure are shortened from seven to five turns, indicating *h*ClpP crystalized in a compact state. This conformation is inactive due to the geometry of the catalytic triad. The H178 residue is oriented away from S153, breaking the hydrogen-bonding network needed for catalysis, and is instead rotated toward D227(Fig. 4C).

Continuous density for **26** (DA29) is evident in the H pocket 2Fo-Fc maps contoured at 1.0σ, with clear composite omit density at 1.8σ, supporting drug binding at the H pocket (Fig. 4D). The H pocket is formed by two adjacent subunits in the apical domain of ClpP. **26** (DA29) adopts a pincer topology similar to **ONC201**, **THPPD** analogs and **ZG111**, with the eastern and western fragments extending into neighboring hydrophobic cavities in the H site (Fig. 4B). The water mediated hydrogen bonding between the piperazine carboxyl and Q107 is present in eight of the fourteen binding pockets. The remaining six **26** (DA29) molecules are oriented in the same geometry, but with little to no electron density for the coordinated water. The interaction between Y118 and the piperazine ring is present in all 14 subunits (Fig. 4E). However, the interaction occurs with the amide nitrogen atom rather than the ring amine hypothesized in Fig 3B.

Both arms of **26** (DA29) feature a trifluoromethyl monosubstituted phenyl ring. The arm with the para substituted ring binds the hydrophobic groove closest to the *h*ClpP entrance pore while the arm with the meta substituted ring binds the groove further from the pore (Fig. 4B), The para substituted arm forms hydrogen bonds between fluorine and the S108 sidechain, as well as the backbone amide of F105 (Fig. 4F). The meta substituted trifluoromethyl group does not appear to participate in any hydrogen bonding. Rather this arm is held in place by π-π interactions with nearby aromatic residues H116, Y118, Y138, and W146. (Fig. 4G).

### Cellular thermal shift assay for drug binding affinity to *h*ClpP

Hydrolytic enzymes such as *h*ClpP are characterized by high structural stability, maintaining catalytic activity even at elevated temperatures (>50 °C) and in the case of lipases also in non-aqueous solvents^20–22^. To assess the interaction of compound **26** (DA29) with *h*ClpP in Caco-2 cells, a cellular thermal shift assay (CETSA) was performed. Cells were incubated with 20 μM of **26** (DA29) for 1 h, and the thermal stability of *h*ClpP was evaluated by monitoring the soluble protein fraction across a temperature gradient using western blotting. Band intensities were normalized to the signal at 55 °C, set as 100%, and expressed as relative percentages for comparison. Treatment with **26** (DA29) markedly enhanced the thermal stability of *h*ClpP compared to the negative control **33** (DA28), confirming intracellular target engagement and supporting the cell-free enzymatic assay results obtained with FITC-casein (Fig. 4H).

### Cytotoxicity in DIPG cell lines with different sensitivity to ONC201 and patient-derived tumor organoids (PDT-O)

Among the synthesized piperazines, only the most potent *h*ClpP activators-**11** (DA24), **15** (DA35), **21** (DA11), **22** (DA14), **23** (DA25), **24** (DA26), **25** (DA23), **26** (DA29), and **33** (DA28) - were tested for antiproliferative activity in two patient-derived DIPG cell lines, SU-DIPG-36 (H3.1K27M) and SU-DIPG-50 (H3.3K27M) (Table 2). The compounds elicited distinct responses in the two models, highlighting differences in drug sensitivity. Notably, **24** (DA26) and **26** (DA29) were the most effective in SU-DIPG-36, with IC_50_ values of 7.8 μM (80% cell death) and 4.0 μM (86% cell death), respectively. In SU-DIPG-50, compound **26** (DA29) retained activity (IC_50_ = 11.6 μM, 69.1% cell death), whereas **24** (DA26) showed a remarkable reduced potency (IC_50_ = 41 μM, 57% cell death). Compound **11** (DA24) was active in both models, with slightly higher efficacy in SU-DIPG-36 (IC_50_ = 55 μM, 82.1% cell death at 100 μM) than in SU-DIPG-50 (IC_50_ = 15 μM, 68.1% cell death at 100 μM). Conversely, **21** (DA11), **23** (DA25), and **33** (DA28) displayed minimal cytotoxicity in both cell line, indicating weak engagement in essential survival pathways. Overall, similar to **ONC201**^2^, the tested compounds showed greater efficacy in SU-DIPG-36 than in SU-DIPG-50 (considering the cell death percentage), underscoring the influence of the molecular subtype on therapeutic response.

To further validate the therapeutic potential of **26** (DA29) in a clinically relevant tumoral context, we assessed its cytotoxic efficacy using DIPG patient-derived tumor organoids (PDT-O). These 3D organoid models, established from fresh biopsies, have been previously characterized and demonstrated to faithfully recapitulate the intratumorally heterogeneity, genetic landscape, and therapeutic responses of their original tumors^23^.

**Figure 5.**
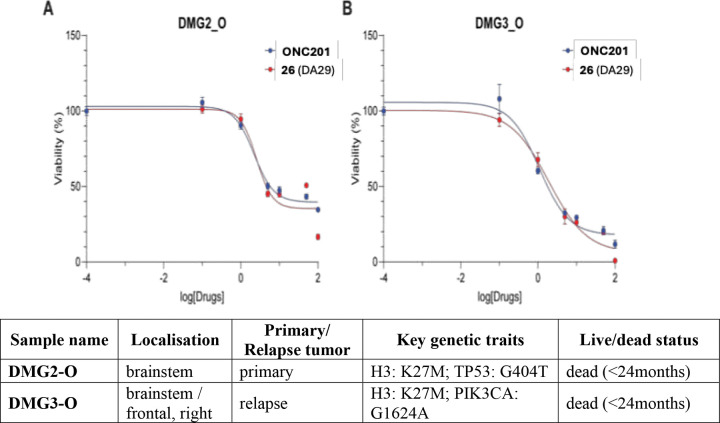
Cytotoxic activity of 26 (DA29) and ONC201 in patient-derived DIPG tumor organoid models. Dose-response curves show the viability of DMG2_O (**A**) and DMG3_O (**B**) following a 72h treatment with **26** (DA29) or **ONC201**. Cell viability was assessed using the CellTiter-Glo^®^ 3D Cell Viability Assay and expressed as percentage of untreated (vehicle) controls (n = 6).

Two distinct PDT-O lines, DMG2_O and DMG3_O, both harboring the H3.3K27M mutation, were treated with increasing concentrations of **26** (DA29) and reference compound **ONC201**. Dose-response curves revealed that **26** (DA29) reduced cell viability similarly to **ONC201** in both PDT-O models (Fig. 5), with IC_50_ value of 2.57 ± 0.04 μM for **26** (DA29) compared to 2.23 ± 0.13 μM for **ONC201** in the DMG2_O model, and 1.94 ± 0.35 μM for **26** (DA29) compared to 1.11 ± 0.18 μM for **ONC201** in the DMG3_O model. Hence, **26** (DA29) demonstrated cytotoxic activity comparable to **ONC201** in clinically pertinent PDT-O models.

### Membrane permeability of ONC201, 26 (DA29), quercetin and caffeic acid

**Figure 6.**
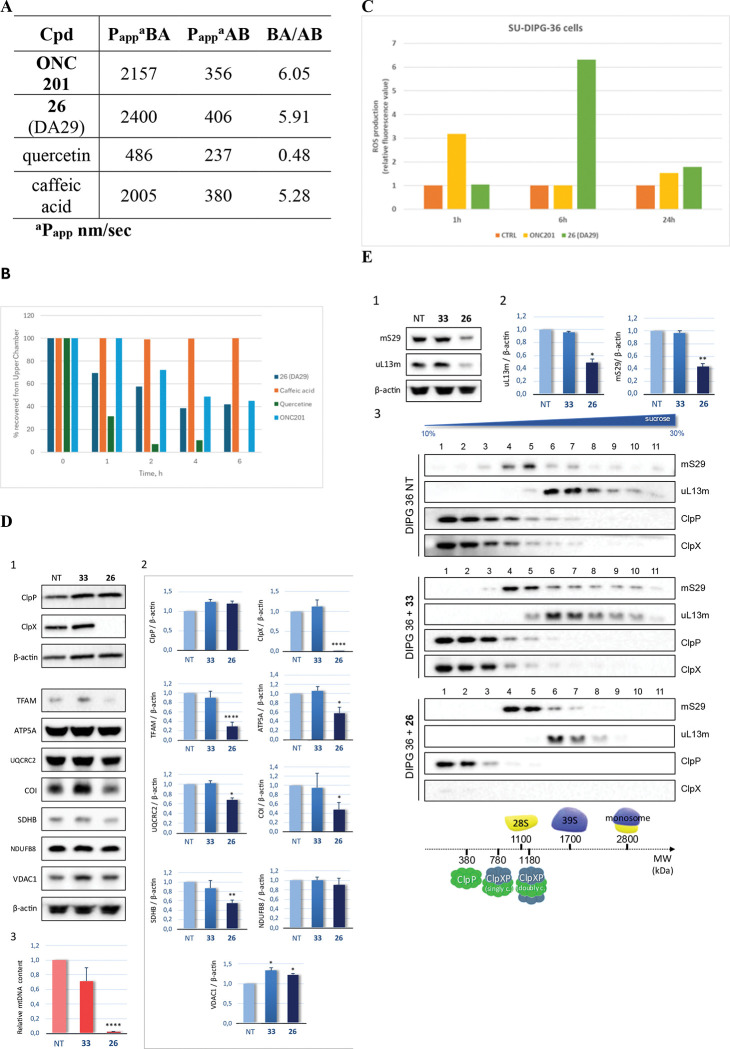
Permeation of 26 (DA29) across the Caco-2 monolayer and Mitochondrial effect of 26 (DA29) treatments on SU-DIPG-36 cells. **A) P**_**app**_ of **ONC201**, **26** (DA29), quercetin and caffeic acid through Caco-2 cell line monolayer. **B**) Time-dependent decrease in the percentage of compound recovered from the apical (upper) chamber under dynamic flow conditions in the FlowCell^®^ LB2 bioreactor. The graph shows the percentage of **26** (DA29) (blue), caffeic acid (orange), and quercetin (green) remaining in the apical compartment at various time points, as determined by HPLC quantification at their respective λ_max_ using calibration curves. **C**) ROS production in the presence of 20 μM **ONC201** or 1 μM **26** (DA29) in SU-DIPG-36 cells. **D**) Mitochondrial effect of **26** (DA29) treatments on SU-DIPG-36 cells. **1)** Whole cell lysates were prepared after 24 hours of treatment with either negative control **33** (DA28) or **26** (DA29) and separated by 12% SDS/PAGE. Immunodetection by specific antibodies revealed the steady-state level of examined proteins (depicted on the left); **2**) bars represent the relative quantification of proteins shown in panel B, normalized to β-actin. Results are presented as the mean ± SEM and are representative of at least three replicates. Statistical analysis was performed using the one-sample Student’s t test (*, p<0.05; **, p<0.01; ****, p<0.0001); **3**) mitochondrial DNA relative levels were measured by qPCR. Bars represent the relative mtDNA content of the treated SU-DIPG-36 compared to untreated cells (NT, set as equal to 1), normalized to 18S rRNA gene (endogenous control). Ratios were calculated according to the Pfaffl’s formula and presented as the mean ± SEM (n=4). Statistical analysis was carried out using the one-sample Student’s t test (****, p<0.0001). **E**) Molecular analysis of mitoribosomal subunits and ClpXP complex. **1)** Mitoribosomal subunit steady-state levels were determined by immunoblotting, as described in Figure 6D1 legend; **2**) Bars represent the relative quantification of proteins shown in panel A, normalized to β-actin. Results are presented as the mean ± SEM and are representative of three replicates. Statistical significance was evaluated by one-sample Student’s t test (*, p<0.05; **, p<0.01); **3**) Isokinetic sucrose gradient analysis of mitoribosomal subunits and ClpXP complex in either SU-DIPG-36 cells untreated (NT) or treated with **33** (DA28) (negative control) and **26** (DA29) (ClpP activator). At the bottom, the ClpP and ClpXP complexes (singly and doubly capped), as well as mtSSU (28S), mtLSU (39S) and monosome, are depicted in order of increasing estimate molecular weight (MW) corresponding to the gradient fractions.

Compound **26** (DA29) was investigated for its membrane permeability, with emphasis on its potential to cross biological barriers. *In silico* analysis using the SWISS ADME platform (BOILED-Egg model, logP, and topological polar surface area)^24^ suggested BBB penetration based on its physicochemical profile and pharmacokinetic properties. This prediction was experimentally validated using Caco-2 cells in static and dynamic assays, well-established *in vitro* models for intestinal absorption and, to some extent, BBB permeability due to their expression of multiple efflux and uptake transporters (Fig. 6A).

The apparent permeability coefficients (P_app_, nm/s) of **ONC201**, **26** (DA29), quercetin, and caffeic acid in both basolateral-to-apical (BA) and apical-to-basolateral (AB) directions are summarized in Fig. 6B. Quercetin displayed symmetric bidirectional permeability (Papp A→B = 486 nm/s; Papp B→A = 237 nm/s; BA/AB = 0.48), consistent with passive diffusion with minimal efflux and its known ability to cross the BBB, confirming its role as a positive control. Conversely, caffeic acid exhibited high apical-to-basolateral permeability (Papp A→B = 2005 nm/s) but strong asymmetry (BA/AB = 5.28), indicative of active efflux and poor net BBB transport, thus serving as a negative control.

**ONC201** and **26** (DA29) showed high BA permeability (2157 and 2400 nm/s, respectively) and substantially lower AB values (356 and 406 nm/s), resulting in ratios of 6.05 and 5.91. Notably, **26** (DA29) demonstrated a modestly higher BA permeability and slightly reduced efflux ratio compared to **ONC201**, suggesting an improved permeability profile that could translate into enhanced bioavailability and tissue penetration. **26** (DA29) does not interact with P-gp efflux pumps exhibiting an EC_50_ > 50 μM in the calcein-AM test assay (data not shown).

### Evaluation of 26 (DA29) transport under physiological flow conditions

Dynamic permeability was assessed in the LB2 bioreactor under flow conditions, and its apical concentration was monitored over time by HPLC. Quantification was carried out at λ_max_ of each compound, using calibration curves. The laminar flow applied (150 μL/min; shear stress ≈ 6×10^−4^ Pa) closely reproduced physiological intestinal dynamics^25^. **26** (DA29) displayed a progressive, time-dependent decrease in apical levels, with ~60% depletion after 6 h, indicative of permeation across the Caco-2 monolayer (Fig. 6B). Quercetin and caffeic acid were included as reference standards due to their well-documented permeability differences^26^. Consistent with expectations, quercetin rapidly disappeared from the apical chamber within 2–4 h, confirming high permeability, whereas caffeic acid remained largely unchanged, indicating poor translocation. These controls validated the assay and positioned **26** (DA29) as having intermediate permeability, between the low-permeable caffeic acid and highly permeable quercetin. This profile supports its potential for central nervous system (CNS) delivery, although additional studies are required to clarify the balance between passive diffusion and transporter-mediated mechanisms^27^.

### Reactive Oxygen Species (ROS) production in SU-DIPG-36 cells treated with ONC201 or 26 (DA29)

To further study the anticancer mechanisms of the most promising compound in SU-DIPG-36 cells, **ONC201** and **26** (DA29) were evaluated for their ability to induce intracellular ROS. **ONC201** was tested at 20 μM, while **26** (DA29) was used at 1 μM, with incubation times of 1h, 6 h, and 24 h. The concentrations were selected according to the different EC_50_ cytotoxicity observed for the two compounds. Intracellular ROS were detected using 1 μM dichloro-dihydro-fluorescein diacetate (DCFH-DA) for 1h. Menadione (25 μM, 50 min) served as a positive control for ROS induction^28^, whereas 80 μM 6-phenethyl-3-(4-(trifluoromethyl)benzyl)-5,6,7,8-tetrahydropyrido[4,3-*d*]pyrimidine-2,4(1*H*,3*H*)-dione (**TH1**) was employed as a negative control (data not shown).

**ONC201** induced a fluorescence increase after 1 h, consistent with ROS production, which returned to basal x-median values (comparable to the control, 1 μM DCFH-DA at 1 h) after 6 h of treatment. **26** (DA29) triggered a marked fluorescence rise at 6 h, followed by a return to baseline at 24 h (Fig. 6C). As expected, **TH1** did not alter fluorescence, confirming its inability to promote ROS generation.

### 26 (DA29) targeted *h*ClpP and hampered mitochondrial function in SU-DIPG-36 cells

Data reported above demonstrated that **26** (DA29) strongly activates purified recombinant *h*ClpP *in vitro*. On this basis, we evaluated whether **26** (DA29) could also bind and hyperactivate the endogenous ClpXP complex in human cells. As previously shown by us^2^ and others^29^, ClpP chemical activators such as imipridones displace the chaperone subunit ClpX from the complex, leading to its degradation through a not fully defined mechanism. ClpX suppression is therefore considered a hallmark of ClpP engagement.

In cultured DIPG cells treated with **26** (DA29) for 24 h at concentrations comparable with the EC_50_ values, ClpX was completely abolished while ClpP remained unaffected (Fig. 6D1). In contrast, the negative control **33** (DA28) had no effect on either protein, confirming that **26** (DA29) specifically targets the ClpXP complex in cells.

The chemo-activation of ClpP promotes uncontrolled degradation of mitochondrial proteins, including respiratory chain subunits, TCA cycle enzymes, and the transcription factor TFAM, as also demonstrated by Mabanglo *et al*^30^. To test whether **26** (DA29) triggered this effect in DIPG cells, we examined representative subunits of the respiratory chain. After 24 h treatment, SDHB, UQCRC2, and ATP5A (Complex II, III, and V, respectively) were markedly reduced, whereas NDUFB8 (Complex I) was unaffected, suggesting that Complex I remained assembled. Moreover, the mitochondrial-encoded Cytochrome C Oxidase subunit I (COI, Complex IV) decreased (Fig. 6D1). These results indicate that ClpP hyperactivation by **26** (DA29) leads to the degradation of respiratory chain components, thereby impairing OXPHOS.

Consistently, **26** (DA29) also caused a dramatic reduction in mtDNA levels (Fig. 6D3). Since mtDNA maintenance depends mainly on TFAM, we measured its expression and found a strong decrease (Fig. 6D1–2). As TFAM is a substrate of activated ClpP, its loss is likely a direct consequence of ClpP hyperactivation. Longer exposures would probably result in complete TFAM suppression but were lethal in SU-DIPG-36 cells. Notably, VDAC1, a marker of mitochondrial mass, remained unchanged, suggesting that mitochondrial biogenesis was not impaired after 24 h of treatment.

### 26 (DA29) negatively affected polysome formation by activating ClpP in cellulo

Mitochondrial ribosomes are a central hub for protein quality control, integrating signals from various mitochondrial processes^31^. Disruption of this system can activate a stress response linking ribosome homeostasis and mitochondrial dysfunction^32^. Given the marked decrease in mtDNA and the degradation of OXPHOS subunits upon **26** (DA29) treatment, we investigated its effects on mitochondrial gene expression, focusing on mitoribosomes proteins (MRPs).

We first measured the steady-state levels of two MRPs, uL13m and mS29, representative proteins of the large (mtLSU, 39S) and small (mtSSU, 28S) ribosomal subunits. As expected, since mtDNA encodes both mt-rRNAs, **26** (DA29) treatment significantly reduced both MRPs (Fig. 6E1–2), indicating fewer ribosomes available for translation.

To assess the mitoribosome assembly in untreated and **33** (DA28) or **26** (DA29)-treated SU-DIPG-36 cells, total proteins were fractionated on an isokinetic sucrose gradient. Analysis of the distribution along the gradient of mS29 and uL13m subunits showed that both mtSSU (fractions 4–5) and mtLSU (fractions 6–7) subunits were correctly assembled in all SU-DIPG-36 cells. However, in **26** (DA29)-treated cells both ribosomal subunits were concurrently undetectable in the highest density fractions (fractions 8–11). This finding, in line with the decrease of both subunits observed above, suggests that when SU-DIPG-36 cells were treated with **26** (DA29) but not with **33** (DA28), normal polysome formation does not occur, which is indicative of inefficient mitochondrial translation (Fig. 6E3).

The same analysis was used to evaluate ClpXP assembly. In untreated and **33** (DA28)-treated cells, ClpP and ClpX co-localized and were also present in high-density fractions (5–7), consistent with intact high-molecular-weight complexes. Upon **26** (DA29) treatment, ClpP was mostly found in the lighter fractions where ClpX was almost undetectable, indicating disassembly of the ClpXP complex and loss of the ClpX hexameric cap (Fig. 6E3). Overall, these findings confirm that **26** (DA29) binds ClpP in cellulo, displaces ClpX, and promotes its degradation.

### Lipid profile in SH-SY5Y and SU-DIPG-36 cell lines treated with ONC201 and 26 (DA29)

Activation of *h*ClpP in cancer cells disrupts several metabolic pathways, including lipid metabolism^33^. To explore this effect, lipidomic profiling of SU-DIPG-36 and SH-SY5Y cells (neuroblastoma cell line from a metastatic bone tumor), untreated or treated with **ONC201** and **26** (DA29) was performed by GC-MS/FID (fatty acids) and HPLC-MS/MS (intact lipids). Lipid composition is one of key indicators of cell health, and treatments with drugs can markedly alter lipid metabolism.

GC-MS analyses identified 34 fatty acids across different chemical classes according to number and position of double bonds along the carbon chain. In untreated cells, saturated fatty acids (SFAs) were most abundant, ranging from 49.99 ± 0.07 % in SH-SY5Y to 68.65 ± 0.16 % in SU-DIPG-36. The main SFAs were myristic (C14:0, ~1.3–1.4 %), palmitic (C16:0, 28.69 ± 0.04 % and 41.15 ± 0.20 % in SH-SY5Y and SU-DIPG-36, respectively), and stearic acid (C18:0, 18.01 ± 0.03 % and 41.15 ± 0.20 %, respectively). Monounsaturated fatty acids (MUFAs) were the second most represented class (40.92 ± 0.16 % in SH-SY5Y; 23.33 ± 0.17 % in SU-DIPG-36), mainly palmitoleic (C16:1ω7, 0.71–3.36 %), oleic (C18:1ω9, 12.77–28.85 %), and vaccenic acid (C18:1ω7, 3.25–5.00 %). Polyunsaturated fatty acids (PUFAs) were least abundant (9.10 ± 0.23 % in SH-SY5Y; 8.01 ± 0.04 % in SU-DIPG-36), with linoleic (C18:2ω6, 0.26–2.46 %), arachidonic (C20:4ω6, 0.37–2.84 %), and docosahexaenoic acid (C22:6ω3, trace-1.41 %) as main representatives (Table S3).

**Figure 7.**
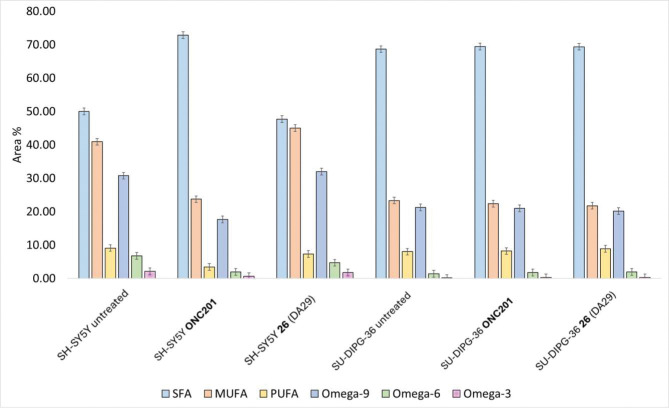
Effect of ONC201 and 26 (DA29) on the lipid content of SFA, MUFA, PUFA, omega-9, omega-6, and omega-3 classes, in SH-SY5Y and SU-DIPG-36 cell lines. For each identified species, the error bars indicate the standard deviation.

As previously reported^2^, drug treatments alter fatty acid composition, particularly MUFAs and PUFAs due to their higher susceptibility to oxidation processes than SFAs, in accordance with the availability of allylic hydrogen. **ONC201** (24 h treatment) markedly reduced MUFA and PUFA levels in SH-SY5Y, suggesting an effect on membrane composition (Fig. 7). By contrast, **26** (DA29) did not significantly affect SU-DIPG-36 cells under the same conditions^2^. Indeed, fatty acid distributions in SU-DIPG-36 remained comparable between untreated and treated samples (Table S3), consistent with earlier findings in this cell line^2^. Therefore, further analyses were extended to intact lipids determined using HPLC-MS/MS.

The untargeted HPLC-MS/MS analysis identified 45 lipid species in SH-SY5Y and 51 in SU-DIPG-36 cells (Tables S4, S5). Lipids were separated according to polarity and eluted by increasing partition number (PN) calculated using the equation PN = CN-2DB, where CN and DB are the carbon and double bond numbers, respectively. The chromatogram was divided into three regions: very polar lipids (LPLs), medium-polar lipids (PLs and SMs), and non-polar lipids (TGs), eluted in the PN range 42–52.

The complete list of molecules, with retention time (tR), detected ions, class, PN, CN, and DB values, is given in Tables S4 and S5. TGs were mainly detected as ammonium and sodium adducts, while PLs appeared as protonated ions. Relative quantification was performed for choline-containing lipids (LPCs, PCs and SMs) and TGs, assuming comparable MS response and recovery during extraction within each lipid class (Tables S4, S5). Differences in the relative abundance of choline-containing lipids and TGs were evaluated by two-tailed t test (p < 0.05) and are reported in Tables S6 and S7, and in Fig. S2 and S3, respectively.

Treatment of SU-DIPG-36 cells with **ONC201** or **26** (DA29) showed similar effects on choline-containing lipids (LPCs) and TGs after 24 h. Almost all LPCs decreased significantly, except LPC 14:0 and 16:1, and most SMs were reduced, except SM 31:0 and SM 34:1. A comparable decrease of all LPCs was observed in SH-SY5Y cells for both drugs (Table S6). This effect may reflect impaired mitochondrial function, since low LPC levels reduce glycerol phosphate dehydrogenase (GPDH) activity^34^, or could represent a defense mechanism against ROS induced by *h*ClpP activation^35^.

For TGs, a significant decrease was observed in species with PN 50 and 52 (TG 52:1, TG 54:2, TG 56:3, TG 54:1) and TG 54:3 (PN 48), while TGs with lower PN (42–48) increased, except TG 52:5, in SU-DIPG-36 cells treated with both compounds. This pattern correlates with previous findings of increased C16 fatty acids (e.g., palmitic acid, C16:0) and decreased long-chain fatty acids in SU-DIPG-36 cells treated with similar drugs^2^. A similar, though less pronounced, trend was also seen in SH-SY5Y cells (Table S7). This behavior could be caused by the inhibition of the very-long-chain fatty acids elongase (ELOVLs) enzymes, responsible for the elongation of shorter chain fatty acids, following the disruption of the mitochondrial function caused by the treatment^36^.

## Conclusion

Through a stepwise rational design strategy, we optimized the piperazine scaffold to enhance interactions within the *h*ClpP hydrophobic allosteric pocket. While piperazine bearing an exocyclic carboxyl markedly reduced activity, incorporation of an endocyclic carboxyl in the 2-oxopiperazine scaffold restored the critical interaction with Q107 and, when combined with electron-withdrawing substituents on both benzyl arms, compound **26** (DA29) was identified as the most potent *h*ClpP activator known to date (EC_50_ = 0.85 ± 0.14 μM; 95% activation), with negligible activity on dopamine D2-like receptors.

High-resolution X-ray crystallography revealed that **26** (DA29) binds in the *h*ClpP H pocket with a characteristic pincer topology, forming a network of hydrogen bonds with Q107, Y118, and S108 and engaging in π-π and anion-π interactions with aromatic residues. These features induce axial pore expansion and displacement of the ClpX co-chaperone, hallmarks of *h*ClpP hyperactivation.

The initial hypotheses for the rational design of piperazines, supported by biological results, were then compared by identifying the actual interactions of **26** (DA29) through its crystallographic structure. The aminic nitrogen atom of **26** (DA29) establishes H-bonding with the hydroxyl of tyrosine (Y118) as in THPPDs. The side chain of glutamine (Q107) participates in an extensive network of hydrogen bonds, forming, with the amide carboxyl function of **26** (DA29), a water-mediated bridge in which the amide backbone of leucine (L104) also participates, confirming once again the structural overlap with THPPDs. The interaction of the second carboxyl function of THPPDs with glutamic acid (E82), which further stabilizes the bond, is also observed in **26** (DA29). In this case, however, an anion-π interaction is observed with the phenyl ring substituted with the *p*-CF3, an interaction made possible by the trifluoromethyl, thanks to its strong electron-withdrawing effect that deactivates the phenyl. This underlines the importance of substituents EWGs in the piperazines, confirming the structural overlap between the different derivatives (Fig. 1).

Consistently, CETSA confirmed intracellular target engagement, while biochemical analyses demonstrated ClpX degradation, loss of OXPHOS subunits and TFAM, mtDNA depletion, and impaired mitoribosome assembly, culminating in defective mitochondrial translation.

Functionally, **26** (DA29) exhibited potent cytotoxicity, with greater efficacy in SU-DIPG-36 than SU-DIPG-50 cell lines, and retained activity in patient-derived organoids, achieving effects comparable to **ONC201**. Importantly, permeability assays in static and dynamic Caco-2 systems demonstrated favorable basolateral transport and reduced efflux compared to **ONC201**, supporting its potential for CNS delivery. Finally, lipidomic profiling revealed consistent remodeling of LPCs and TGs, corroborating mitochondrial dysfunction and suggesting metabolic signatures as biomarkers of *h*ClpP hyperactivation.

Together, these findings establish **26** (DA29) as a new generation of *h*ClpP activator with optimized potency, selectivity, and pharmacokinetic properties. By dismantling mitochondrial proteostasis and bioenergetics, **26** (DA29) exerts robust anticancer activity in translational DIPG models, thereby providing a promising lead compound for therapeutic development in paediatric brain tumors and other *h*ClpP-dependent malignancies.

### Experimental Section

#### Chemistry.

^1^H NMR and ^13^C NMR spectra were recorded on a Bruker 600 MHz or AGILENT 500 MHz spectrometer and chemical shifts are reported in parts per million (δ), and the following abbreviations were used to explain the multiplicities: s = singlet, d = doublet, dt = doublet of triplet, t = triplet, td = triplet of doublet, q = quartet, m = multiplet, quin = quintuplet, sext = sextet, sep = septet, b = broad. FT-IR spectra were recorded on a Perkin-Elmer 681 spectrophotometer. GC analyses were performed on a HP 6890 model, Series II by using a HP1 column (methyl siloxane; 30 m × 0.32 mm × 0.25 μm film thickness). Analytical thin-layer chromatography (TLC) was carried out on pre-coated 0.25 mm thick plates of Kieselgel 60 F254; visualization was accomplished by UV light (254 nm). Column chromatography was accomplished by using silica gel 60 with a particle size distribution 40–63 μm and 230–400 ASTM. GC-MS analyses were performed on HP 5995C model. High-resolution mass spectrometry (HRMS) analyses were performed using a Bruker microTOF QII mass spectrometer equipped with an electrospray ion source (ESI). Reagents and solvents were purchased from Sigma-Aldrich (Sigma-Aldrich, St. Louis, MO, USA) and used without any further purification. Full characterization data have been reported for both the newly synthesized compounds and the known compounds. All spectral data are consistent with the reported values.

#### General synthesis of 1–2.

To a stirred suspension of piperazine (5.80 mmol) and K_2_CO_3_ (14.5 mmol) in CH_3_CN (14 mL) was added the appropriate benzyl bromide (12.76 mmol). The reaction mixture was stirred at room temperature for 2 h. The reaction progress was monitored by TLC (CH_2_Cl_2_/MeOH = 9.8/0.2 as mobile phase). Then, the resulting solid was filtered and repeatedly washed with EtOAc. The organic filtrate was concentrated under reduced pression, and the resulting brown semisolid underwent to chromatography on silica gel and CH_2_Cl_2_/MeOH = 9.8/0.2 as mobile phase for **1** and CH_2_Cl_2_/MeOH = 9.5/0.5 as the mobile phase for **2**.

#### 1,4-Bis(4-chlorobenzyl)piperazine 1 (DA1).

White solid, 25% yield. M.p. 136–138 °C. ^1^H NMR (500 MHz, CDCl_3_, δ): 7.29–7.22 (m, 8H, aromatic protons), 3.47 (s, 4H, 2Ph*CH*_*2*_N), 2.46 (s, 8H, N*(CH*_*2*_*)*_*2*_N*(CH*_*2*_*)*_*2*_). ^13^C NMR (125 MHz, CDCl_3_, δ): 136.28, 132.48, 130.16, 128.07, 61.90, 52.65. GC−MS (70 eV) (*m/z*) (rel. int.) 335 (M^+^, 2), 334 (8), 209 (33), 180 (5), 166 (6), 154 (21), 125 (100), 89 (14), 42 (6). HPLC: Rt = 14.023 min (mobile phase: CH_3_CN/CH_3_COONH_4_ = 70/30; stationary phase: ZORBAX ECLIPSE Plus C18, analytical 4.6×250mm, 5 μm); rate = 1mL/min.

#### 1,4-Bis(4-cyanobenzyl)piperazine 2 (DA4).

White solid, 39% yield. M.p. 127–130 °C. ^1^H NMR (500 MHz, CDCl_3_, δ): 7.65 (s, 2H, aromatic protons), 7.56–7.53 (m, 4H, aromatic protons), 7.41 (t, 2H, *J* = 7.7 Hz, aromatic protons), 3.54 (s, 4H, 2NC*H*_*2*_Ph), 2.49 (s, 8H, N*(CH*_*2*_*)*_*2*_N*(CH*_*2*_*)*_*2*_). ^13^C NMR (125 MHz, CDCl_3_, δ): 139.81, 133.36, 132.41, 130.85, 129.05, 118.89, 112.39, 61.89, 52.89. GC−MS (70 eV) (*m/z*) (rel. int.) 316 (M^+^,10), 200 (34), 171 (13), 157 (16), 145 (52), 116 (100), 89 (21), 56 (9), 42 (12). HPLC: Rt = 5.058 min (mobile phase: CH_3_CN/CH_3_COONH_4_ = 70/30; stationary phase: ZORBAX ECLIPSE Plus C18, analytical 4.6×250mm, 5 μm); rate = 1mL/min.

#### 3-(Piperazin-1-ylmethyl)benzonitrile 3 (DA2).

To a stirred suspension of piperazine (0.351 g; 4.08 mmol) and K2CO3 (0.169 g; 1.22 mmol) in CH_3_CN (10 mL) was added the 3-(Bromomethyl)benzonitrile (0.200g; 1.02 mmol). The reaction mixture was stirred at room temperature for 2 h. The reaction progress was monitored by TLC (CH_2_Cl_2_/MeOH = 9.5/0.5 as mobile phase). Then, the resulting solid were filtered and repeatedly washed with EtOAc. The organic filtrate was concentrated under reduced pression, and the product was isolated by purification with chromatography on silica gel and CH_2_Cl_2_/MeOH = 9/1 as the mobile phase. White semisolid, 81% yield. ^1^H NMR (500 MHz, DMSO, δ): 7.70–7.67 (m, 2H, aromatic protons), 7.63–7.59 (m, 1H, aromatic proton), 7.53–7.48 (m, 1H, aromatic proton), 3.45 (s, 2H, NCPhC*H*_*2*_N), 2.66 (t, 4H, *J* = 4.8 Hz, 2NHC*H*_*2*_), 2.32–2.18 (m, 4H, 2NC*H*_*2*_). GC−MS (70 eV) (*m/z*) (rel. int.) 201 (M^+^, 17), 159 (100), 116 (79), 89 (16), 56 (22), 42 (1).

#### 1-(3-Cyanobenzyl)-4-(3-(trifluoromethyl)benzyl)piperazine 4 (DA7)*.*

To a stirred suspension of **3** (0.196 g; 0.98 mmol) and K_2_CO_3_ (0.175g; 1.27 mmol) in CH_3_CN (5 mL) was added 3-(Trifluoromethyl)benzyl bromide (0.16 ml; 1.073 mmol). The reaction progress was monitored by TLC (EtOAc/hexane = 3/7 as mobile phase). Then, the resulting solid was filtered and repeatedly washed with EtOAc. The organic filtrate was concentrated under reduced pression, and the crude residue was purified through column chromatography (silica gel, EtOAc/hexane = 3/7 as mobile phase). Orange oil; 59% yield. ^1^H NMR (500 MHz, CDCl_3_, δ): 7.65 (s, 1H, aromatic proton), 7.59 (s, 1H, aromatic proton), 7.57–7.50 (m, 4H, aromatic protons), 7.42 (dd, 2H, *J*_*1*_ = 9.15 Hz, *J*_*2*_ = 7.75 Hz, aromatic protons), 3.58 (s, 2H, NC*H*_*2*_PhCF3), 3.54 (s, 2H, NC*H*_*2*_PhCN), 2.51 (s, 8H, N*(CH*_*2*_*)*_*2*_N*(CH*_*2*_*)*_*2*_). ^13^C NMR (125 MHz, CDCl_3_, δ): 133.40, 132.46, 132.44, 130.87, 130.63 (q, *J* = 32 Hz), 129.06, 128.73, 125.72 (q, *J* = 3.2 Hz), 124.65 (q, *J* = 270.7 Hz), 124.09 (q, *J* = 3.5 Hz), 118.87, 112.40, 62.19, 61.85, 52.79. GC−MS (70 eV) (*m/z*) (rel. int.) 359 (M^+^, 25), 243 (29), 200 (51), 188 (49), 159 (100), 145 (33), 116 (77), 89 (17),56 (11), 42 (16). HPLC Rt = 5.058 min (mobile phase: CH_3_CN/CH_3_COONH_4_ 70/30; stationary phase: ZORBAX ECLIPSE Plus C18, analytical 4.6×250mm, 5 μm); rate = 1mL/min.

#### General synthesis of 5–8.

To a solution of piperazine (4.98 mmol) in absolute EtOH (3 mL), was added the appropriate benzoyl chloride (2.49 mmol), at 0 °C. The reaction mixture was stirred at room temperature, overnight. The reaction progress was monitored by TLC (CH_2_Cl_2_/MeOH = 9/1 as mobile phase). The solvent was evaporated under reduced pressure and to the residue was added CH_2_Cl_2_. The organic layer was washed with brine and was dried on anhydrous Na_2_SO_4_. The crude residue was purified by column chromatography (silica gel and CH_2_Cl_2_/MeOH = 9/1 as the mobile phase).

#### 1-Benzoyl piperazine 5 (DA13).

White solid, 19% yield. M.p. 62–65 °C. ^1^H NMR (300 MHz, CDCl_3_, δ): 7.44–7.32 (m, 5H, aromatic protons), 4.69–4.27 (m, 8H, CON(C*H*_*2*_)_*2*_, NH(C*H*_*2*_)_*2*_). GC−MS (70 eV) (*m/z*) (rel. int.) 190 (M^+^, 9), 160 (8), 134 (22), 122 (15), 105 (100), 85 (51), 77 (63), 69 (96), 56 (71), 51 (21), 42 (11).

#### 3-(Piperazine-1-carbonyl)benzonitrile 6 (DA16).

White solid, 3% yield. M.p. 164–167 °C. ^1^H NMR (300 MHz, CDCl_3_, δ): 7.73–7.66 (m, 2H, aromatic protons), 7.63 (dt, 1H, *J*_*1*_ = 7.8 Hz, *J*_*2*_ = 1.41 Hz, aromatic proton), 7.57–7.49 (m, 1H, aromatic proton), 3.74 (bs, 2H, CONC*H*_*2*_), 3.36 (bs, 2H, CONC*H*_*2*_), 3.01–2.71 (m, 4H, C*H*_*2*_NHC*H*_*2*_), 1.99 (s, 1H, N*H*). GC−MS (70 eV) (*m/z*) (rel. int.) 215 (M^+^, 9), 185 (14), 130 (83), 102 (46), 85 (36), 75 (13), 69 (71), 56 (100), 42 (15).

#### 1,4-Dibenzoylpiperazine 7 (DA12).

White solid, 4% yield. M.p. 190–193 °C. ^1^H NMR (300 MHz, CDCl_3_, δ): 7.49–7.36 (m, 10H, aromatic protons), 3.63 (bs, 8H, N*(CH*_*2*_*)*_*2*_N*(CH*_*2*_*)*_*2*_).^13^C NMR (125 MHz, CDCl_3_, δ): 170.64, 135.11, 130.08, 128.63, 127.04, 47.61, 42.35. GC−MS (70 eV) (*m/z*) (rel. int.) 294 (M^+^, 3), 226 (9), 105 (100), 77 (39), 69 (11), 51 (8). HPLC Rt = 3.010 min (mobile phase: CH_3_CN/H_2_O 70/30; stationary phase: ZORBAX ECLIPSE Plus C18, analytical 4.6×250mm, 5 μm); rate = 1mL/min.

#### 3,3’-(Piperazine-1,4-dicarbonyl)dibenzonitrile 8 (DA15)*.*

White solid, 22% yield. M.p. 212–215 °C. ^1^H NMR (500 MHz, DMSO, δ): 8.01–7.86 (m, 4H, aromatic protons), 7.81–7.72 (m, 2H, aromatic protons), 7.70–7.59 (m, 2H, aromatic protons), 3.82–3.56 (m, 4H, 2CONC*H*_*2*_), 3.49–3.26 (m, 4H, 2CONCH_2_C*H*_*2*_). ^13^C NMR (125 MHz, DMSO, δ): 167.69, 137.28, 137.26, 133.79, 132.21, 132.19, 131.05, 130.32, 118.70, 112.11, 47.42, 47.08, 42.19, 42.76. GC−MS (70 eV) (*m/z*) (rel. int.) 344 (M^+^, 0.7), 130 (100), 102 (46), 69 (12), 56 (17), 42 (3).

#### 1-(3-Cyanobenzoyl)-4-(4-chlorobenzoyl)piperazine 9 (DA17).

To a solution of **6** (0.175 g; 0.81 mmol) in absolute EtOH (3 mL) was added 4-chlorobenzoyl chloride (0.214 g, 1.22 mmol), at 0 °C. The reaction mixture was stirred at room temperature, overnight. The reaction progress was monitored by TLC (EtOAc/hexane = 8/2 as mobile phase). The solvent was evaporated under reduced pressure and to the residue was added CH_2_Cl_2_. The organic layer was washed with brine and was dried on anhydrous Na_2_SO_4_. The solvent was evaporated under reduced pressure. The product was isolated by column chromatography (silica gel and EtOAc/hexane = 8/2 as the mobile phase). White solid, 43% yield. M.p. 169–172 °C. ^1^H NMR (300 MHz, CDCl_3_, δ): 7.74 (dt, 1H, *J*_*1*_ = 7.5 Hz, *J*_*2*_ = 1.4 Hz, aromatic proton), 7.72–7.69 (m, 1H, aromatic proton), 7.65 (dt, 1H, *J*_*1*_ = 7.8 Hz, *J*_*2*_ = 1.4 Hz, aromatic proton), 7.57 (t, 1H, *J* = 7.68 Hz, aromatic proton), 7.45–7.33 (m, 4H, aromatic protons), 3.64 (bs, 8H, CON(C*H*_*2*_C*H*_*2*_*)*_*2*_NCO). ^13^C NMR (125 MHz, CDCl_3_, δ): 169.59, 168.17, 136.43, 136.33, 133.57, 133.15, 131.31, 130.75, 129.70, 128.99, 128.64, 117.73, 113.19, 47.53, 42.53. GC−MS (70 eV) (*m/z*) (rel. int.) 355 (2), 354 (3), 353 (M^+^, 5), 214 (11), 141 (34), 140 (8), 139 (100), 130 (75), 11 (32), 102 (31), 69 (35), 56 (18), 42 (6). HPLC Rt = 3.236 min (mobile phase: CH_3_CN/CH_3_COONH_4_ 70/30; stationary phase: ZORBAX ECLIPSE Plus C18, analytical 4.6×250mm, 5 μm); rate = 1mL/min.

#### General synthesis of 10–16.

A suspension of 2-oxopiperazine (0.99 mmol) and NaH (1.49 mmol) in anhydrous DMF (3 mL) was stirred at 0 °C for 30 minutes. After this time, the appropriate substituted-benzyl bromide (1.19 mmol) was added to the reaction mixture, and the reaction was heated to 90 °C for 16 h. The reaction mixture was cooled to room temperature, and it was concentrated under reduced pressure. EtOAc was added to residue and the solution was washed with brine. The organic layer was dried on anhydrous Na_2_SO_4_, and the solvent was evaporated under reduced pressure. The resulting crude mixture underwent column chromatography on silica gel to give 10−16.

#### 3,3’-((2-Oxopiperazine-1,4-diyl)bis(methylene))dibenzonitrile 10 (DA9)*.*

Orange oil; 38% yield. ^1^H NMR (500 MHz, CD_3_OD, δ): 7.71 (s, 1H, aromatic proton), 7.68–7.57 (m, 5H, aromatic protons), 7.51 (dt, 2H, *J*_*1*_ = 7.7 Hz, *J*_*2*_ = 2.7 Hz, aromatic protons), 4.63 (s, 2H, NCPhC*H*_*2*_NCO), 3.65 (s, 2H, NCPhC*H*_*2*_NCH_2_), 3.33 (t, 2H, *J* = 5.35 Hz, CONCH_*2*_CH_2_N), 3.23 (s, 2H, NCOC*H*_*2*_N), 2.72 (t, 2H, *J* = 5.55 Hz, NC*H*_*2*_CH_2_NCO). ^13^C NMR (125 MHz, CD_3_OD, δ): 167.97, 138.89, 138.38, 133.53, 132.38, 132.28, 131.19, 131.05, 131.02, 129.51, 129.28, 59.93, 56.37, 48.65, 48.55, 46.24. HRMS (ESI) *m/z*: calc. for [C_18_H_18_Br_2_N_2_O + H]^+^: 436.9840; ESI-MS-MS: 436.9840, 410.9855, 212.0059, 168.9639, 90.0462. ESI-MS *m/z*: [C_20_H_18_N_4_O + Na]^+^: 353.1374; ESI-MS-MS: 353.1378, 170.9956, 139.0394, 121.0367, 76.9972. ESI-MS *m/z*: [C_20_H_18_N_4_O−H]^−^, 329.1424. ESI-MS-MS: 329.1417, 143.0622, 116.0510, 70.0302. The compound was purified through column chromatography (silica gel, CH_2_Cl_2_/MeOH = 9.0/0.2 as the mobile phase).

#### 1,4-Bis(3-(Trifluoromethyl)benzyl)piperazin-2-one 11 (DA24).

Orange oil; 37% yield. ^1^H NMR (300 MHz, CDCl_3_, δ): 7.63–7.40 (m, 8H, aromatic protons), 4.65 (s, 2H, CF_3_PhC*H*_*2*_NCO), 3.62 (s, 2H, CF_3_PhC*H*_*2*_NCH_2_), 3.31–3.19 (m, 4H, NCOC*H*_*2*_N, CONC*H*_*2*_CH_2_N), 2.68 (t, 2H, *J* = 5.2 Hz, NC*H*_*2*_CH_2_NCO). ^13^C NMR (125 MHz, CD_3_OD, δ): 167.24, 157.91, 137.78, 137.26, 132.75, 131.57, 131.56, 131.42, 131.41, 130.81, 130.73, 130.63, 130.56, 130.48, 130.38, 130.30, 129.36, 129.33, 129.21, 129.05, 128.95, 125.55 (q, *J* = 3.85 Hz), 125.24, 125.19, 124.64, 124.62, 124.55, 124,54, 124.38, 124.37, 124.36, 124.33, 124.29, 124.27, 124.07 (q, *J* = 3.85 Hz), 123.09, 123.04, 60.13, 55.86, 48.71, 48.61, 45.66. GC−MS (70 eV) (*m/z*) (rel. int.) 417 (3), 416 (M^+^, 12), 388 (5), 387 (22), 258 (9), 257 (64), 160 (9), 159 (100), 141 (6), 140 (3), 139 (2), 109 (15), 42 (20). HPLC Rt = 7.937 min (mobile phase: CH_3_CN/CH_3_COONH_4_ 70/30; stationary phase: ZORBAX ECLIPSE Plus C18, analytical 4.6×250mm, 5 μm); rate = 1mL/min. The product was isolated by column chromatography (silica gel and EtOAc/hexane = 7/3 as the mobile phase)

#### 1,4-Bis(2-fluorobenzyl)piperazin-2-one 12 (DA32).

Orange semisolid; 22% yield. ^1^H NMR (500 MHz, CD_3_OD, δ): 7.55–7.39 (m, 1H, aromatic protons), 7.37–7.22 (m, 3H, aromatic protons), 7.16 (t, 1H, *J* = 7.5 Hz, aromatic proton), 7.11 (td, 1H, *J*_*1*_ = 7.5 Hz, *J*_*2*_ = 0.9 Hz aromatic proton), 7.09–7.00 (m, 2H, aromatic protons), 4.65 (s, 2H, FPhC*H*_*2*_NCO), 3.81 (s, 2H, FPhC*H*_*2*_NCH_2_), 3.53–3.26 (m, 4H, NCOC*H*_*2*_N, CONC*H*_*2*_CH_2_N), 2.97–2.72 (m, 2H, CONCH_2_C*H*_*2*_N). ^13^C NMR (125 MHz, CDCl_3_, δ): 161.36 (d, *J* = 245.8 Hz), 161.11 (d, *J* = 2 44.8 Hz), 130.73 (d, *J* = 3.9 Hz), 129.46 (d, *J* = 8.3 Hz), 124.53 (d, *J* = 3.5 Hz), 115.59 (d, *J* = 21.9 Hz), 115.32 (d, *J* = 21.7 Hz), 53.66, 53.63, 48.80, 43.04, 43.01. GC−MS (70 eV) (*m/z*) (rel. int.) 317(2), 316 (M^+^, 12), 288 (4), 287 (18), 208 (6),207 (49), 110 (8), 109 (100), 107 (2), 83 (11), 42 (11). HPLC Rt = 4.515 min (mobile phase: CH_3_CN/CH_3_COONH_4_ 70/30; stationary phase: ZORBAX ECLIPSE Plus C18, analytical 4.6×250mm, 5 μm); rate = 1mL/min. The product was isolated by column chromatography (silica gel and EtOAc/hexane = 1/1 as the mobile phase)

#### 1,4-Bis(4-fluorobenzyl)piperazin-2-one 13 (DA36).

White solid, 40% yield. M.p. 133–137 °C. ^1^H NMR (500 MHz, CDCl_3_, δ): 7.29–7.19 (m, 4H, aromatic protons), 7.04–6.97 (m, 4H, aromatic protons), 4.55 (s, 2H, PhC*H*_*2*_NCO), 3.51 (s, 2H, PhC*H*_*2*_NCH_2_), 3.24–3.17 (m, 4H, NCOC*H*_*2*_N, CONC*H*_*2*_CH_2_N), 2.62 (t, 2H, *J* = 5.45 Hz, CONCH_2_C*H*_*2*_N). ^13^C NMR (125 MHz, CDCl_3_, δ): 166.85, 161.24 (d, *J* = 244.5 Hz), 132.32 (d, *J* = 3.2 Hz), 130.57 (d, *J* = 7.9 Hz), 129.89 (d, *J* = 8.1 Hz), 115.51 (d, *J* = 21.5 Hz), 115.29 (d, *J* = 21.2 Hz), 60.97, 57.24, 49.15, 48.78, 45.87. GC−MS (70 eV) (*m/z*) (rel. int.) 316 (M^+^, 12), 287 (11), 207 (30), 109 (100), 83 (9), 42 (7). HPLC Rt = 4.660 min (mobile phase: CH_3_CN/H_2_O 70/30; stationary phase: ZORBAX ECLIPSE Plus C18, analytical 4.6×250mm, 5 μm); rate = 1mL/min. The product was isolated by column chromatography (silica gel and EtOAc/hexane = 1/1 as the mobile phase)

#### 1,4-Bis(2-bromobenzyl)piperazin-2-one hydrochloride 14 (DA33).

White solid, 6% yield. M.p. 221–225 °C. ^1^H NMR (500 MHz, CD3OD, δ): 7.77 (dd, 1H, *J*_*1*_ = 8.05 Hz, *J*_*2*_ = 1.15 Hz, aromatic proton), 7.74 (dd, 1H, *J*_*1*_ = 7.65 Hz, *J*_*2*_ = 1.65 Hz, aromatic proton), 7.64–7.60 (m, 1H, aromatic proton), 7.52 (td, 1H, *J*_*1*_ = 7.55 Hz, *J*_*2*_ = 1.25 Hz, aromatic proton), 7.43 (td, 1H, *J*_*1*_ = 7.9 Hz, *J*_*2*_ = 1.7 Hz, aromatic proton), 7.40–7.34 ( m, 2H, aromatic protons), 7.27–7.20 (m, 1H, aromatic proton), 4.79 (s, 2H, BrPhC*H*_*2*_NCO), 4.65 (s, 2H, BrPhC*H*_*2*_NCH_2_CH_2_), 4.08 (s, 2H, NCOC*H*_*2*_N), 3.72 (t, 2H, *J* = 5.3 Hz, CONC*H*_*2*_CH_2_N), 3.61 (t, 2H, *J* = 5.8 Hz, CONCH_2_C*H*_*2*_N). ^13^C NMR (125 MHz, CD_3_OD, δ): 161.81, 134.07, 133.59, 133.13, 132.81, 132.02, 129.39, 129.18, 128.75, 128.42, 127.78, 125.57, 123.06, 59.29, 52.61, 49.49, 42.86. HRMS (ESI) *m/z*: calc. for [C_18_H_18_Br_2_N_2_O + H]^+^: 436.9840; ESI-MS-MS: 436.9840, 410.9855, 212.0059, 168.9639, 90.0462. ESI-MS m/z: [C_18_H_18_Br_2_N_2_O + Na]^+^: 460.9645; ESI-MS-MS: 460.9612, 406.2976, 308.9732, 253.0129, 170.9620, 91.0534. HPLC Rt = 4.392 min (mobile phase: CH_3_CN/CH_3_COONH_4_ 90/10; stationary phase: ZORBAX ECLIPSE Plus C18, analytical 4.6×250mm, 5 μm); rate = 1mL/min. The product was crystallized by hot/cold CH_3_OH.

#### 1,4-Bis(4-bromobenzyl)piperazin-2-one 15 (DA35).

Orange oil; 16% yield. ^1^H NMR (500 MHz, CDCl_3_, δ): 7.44 (dd, 4H, *J*_*1*_ = 8.3 Hz, *J*_*2*_ = 1.5 Hz, aromatic protons), 7.18 (d, 2H, *J* = 8.3 Hz, aromatic protons), 7.13 (d, 2H, *J* = 8.3 Hz, aromatic protons), 4.53 (s, 2H, BrPhC*H*_*2*_NCO), 3.50 (s, 2H, BrPhC*H*_*2*_NCH_2_), 3.22 (s, 2H, NCOC*H*_*2*_N), 3.19 (t, 2H, *J* = 5.4 Hz, CONC*H*_*2*_CH_2_N), 2.61 (t, 2H, *J* = 5.35 Hz, CONCH_2_C*H*_*2*_N). ^13^C NMR (125 MHz, CDCl_3_, δ): 166.82, 135.56, 131.78, 131.59, 129.91, 130.64, 61.01, 57.27, 49.14, 48.92, 45.96. GC−MS (70 eV) (m/z) (rel. int.) 439(4),438 (M^+^, 2), 437 (9), 410 (4), 409 (1),408 (6), 269 (39),268 (5), 267 (41),172 (8), 171 (97), 170 (8), 169 (100), 91 (8), 90 (45), 89 (30), 63 (5), 42 (25). HPLC Rt = 5.670 min (mobile phase: CH_3_CN/H_2_O 70/30; stationary phase: ZORBAX ECLIPSE Plus C18, analytical 4.6×250mm, 5 μm); rate = 1mL/min. The product was isolated by column chromatography (silica gel and EtOAc/hexane = 1/1 as the mobile phase).

#### 1,4-Bis(4-chlorobenzyl)piperazin-2-one 16 (DA53).

White solid, 21% yield. M.p 134–137 °C. ^1^H NMR (500 MHz, CDCl_3_, δ): 7.46–7.35 (m, 4H, aromatic protons), 7.28–7.22 (m, 4H, aromatic protons), 4.84 (s, 4H, 2ClPhC*H*_*2*_N), 3.46 (s, 4H, NCOC*H*_*2*_, NCH_*2*_CH_2_), 2.17 (s, 2H, NCH_2_C*H*_*2*_). ^13^C NMR (125 MHz, CDCl_3_, δ): 157.49, 133.84, 132.98, 130.69, 129.74, 129.49, 127.47, 47.92, 44.02, 30.96, 29.69. HRMS (ESI) *m/z*: calc. for ESI-MS *m/z*: [C_18_H_18_Cl_2_N_2_O + Na]^+^: 371.2749; ESI-MS-MS: 371.2749, 342.1111, 265.1137, 212.4230, 185.0124, 76.9972, 62.9828. HPLC Rt =4.178 min (fase mobile: H_2_O/CH_3_CN 30:70; fase stazionaria: ZORBAX ECLIPSE Plus C18, analytical 4.6×250mm, 5-Micron); flusso = 1mL/min. The product was isolated by column chromatography (silica gel and EtOAc/hexane = 6/4 as the mobile phase).

#### General synthesis 17–20.

A mixture of 2-oxopiperazine (0.99 mmol) in anhydrous DMF (3 mL), NaHCO_3_ (1.19 mmol), and the appropriate substituted-benzyl bromide (1.19 mmol) was stirred at 100 °C for 16 h. The reaction mixture was cooled to room temperature, and the solvent was removed under reduced pressure. CH_2_Cl_2_ was added to the reaction crude, and the solution was washed with brine. The organic layer was dried on anhydrous Na_2_SO_4_, and the solvent was evaporated under reduced pressure. The products were isolated by column chromatography (silica gel and EtOAc/MeOH = 9.8/0.2 as the mobile phase).

#### 3-((3-Oxopiperazin-1-yl)methyl)benzonitrile 17 (DA10)*.*

White solid, 56% yield. M.p. 148–151°C. ^1^H NMR (300 MHz, CDCl_3_, δ): 7.65 (m, 1H, aromatic proton), 7.59–7.53 (m, 2H, aromatic protons), 7.44 (dd, 1H, *J*_*1*_ = 8.01 Hz, *J*_*2*_ = 7 Hz, aromatic proton), 6.69 (bs, 1H, NH), 3.60 (s, 2H, NCPhC*H*_*2*_N), 3.41–3.32 (m, 2H, CONHC*H*_*2*_CH_2_N), 3.13 (s, 2H, HNCOC*H*_*2*_N), 2.65 (t, 2H, *J* = 5.52 Hz, CONHCH_2_C*H*_*2*_N). HRMS (ESI) *m/z*: calc. for ESI-MS *m/z*: [C_12_H_13_N_3_O + Na]^+^: 238.0955; ESI-MS-MS: 238.0954, 201.6492, 183.1700, 149.0149, 100.1089, 76.9968, 62.9809. ESI-MS m/z: [C_12_H_13_N_3_O−H]^−^, 214.0995. ESI-MS-MS: 214.0992, 141.0459, 116.0513, 102.0352, 58.0300.

#### 4-(4-Nitrobenzyl)piperazin-2-one 18 (DA22)*.*

White solid, 53% yield. M.p. 152–155 °C. ^1^H NMR (300 MHz, CDCl_3_, δ): 8.24–8.18 (m, 1H, aromatic proton), 8.18–8.08 (m, 1H, aromatic proton), 7.66–7.52 (m, 2H, aromatic protons), 6.78 (bs, 1H, NH), 3.68 (s, 2H, NO_2_PhC*H*_*2*_N), 3.41–3.34 (m, 2H, CONHC*H*_*2*_CH_2_N), 3.16 (s, 2H, NHCOC*H*_*2*_), 2.68 (t, 2H, *J* = 5.5 Hz, CONHCH_2_C*H*_*2*_N). GC−MS (70 eV) (*m/z*) (rel. int.) 235 (M^+^, 16), 206 (15), 165 (33), 136 (64), 99 (100), 90 (42), 42 (66).

#### 4-(3-(Trifluoromethyl)benzyl)piperazin-2-one 19 (DA46).

White solid, 46% yield. M.p. 88–90 °C. ^1^H NMR (300 MHz, CDCl_3_, δ): 7.63–7.39 (m, 4H, aromatic protons), 6.74 (bs, 1H, NH), 4.56 (s, 2H, CF_3_PhC*H*_*2*_N), 3.20–3.18 (m, 4H, COC*H*_*2*_N, CONHC*H*_*2*_CH_2_N), 2.67 (t, 2H, *J* = 5.2 Hz, CONHCH_2_C*H*_*2*_N).

#### 4-(4-(Trifluoromethyl)benzyl)piperazin-2-one (20) (DA40).

White solid, 58% yield. M.p. 119–122 °C. ^1^H NMR (500 MHz, CDCl_3_, δ): 7.58 (d, 2H, *J* = 8 Hz, aromatic protons), 7.45 (d, 2H, *J* = 8 Hz, aromatic protons), 7.26 (bs, NH), 3.63 (s, 2H, CF_3_PhC*H*_*2*_N), 3.40–3.31 (m, 2H, CONHC*H*_*2*_CH_2_), 3.16 (s, 2H, COC*H*_*2*_N), 2.64 (t, 2H, *J* = 5.4 Hz, CONHCH_2_C*H*_*2*_N). GC−MS (70 eV) (*m/z*) (rel. int.) 259 (M^+^, 43), 229 (20), 188 (30), 159 (100), 99 (68), 42 (41).

#### General synthesis of 21–27.

To suspension of NaH (60% dispersion in oil, 0.8 mmol) in anhydrous DMF (1 mL), was added dropwise a solution of **17**-**20** (0.4 mmol) in anhydrous DMF (2 mL), at 0 °C. The reaction mixture is left stirring at 0 °C for 30 minutes. The appropriate benzyl bromide (0.47 mmol) was added at the suspension and the reaction was left, under stirring, at 100 °C, overnight. The reaction mixture was cooled to room temperature and H_2_O and EtOAc were added. The organic phase was washed with brine (x3) and dried over anhydrous Na_2_SO_4_. The solvent was evaporated under reduced pressure to give **21**−**27**.

#### 4-(3-Cyanobenzyl)-1-(4-chlorobenzyl)piperazin-2-one 21 (DA11).

White solid, 43% yield. M.p. 109–111 °C. ^1^H NMR (500 MHz, CDCl_3_, δ): 7.64 (s, 1H, aromatic proton), 7.58–7.55 (m, 2H, aromatic protons), 7.43 (t, 1H, *J* = 7.7 Hz, aromatic proton), 7.31–7.19 (m, 4H, aromatic protons), 4.56 (s, 2H, ClPhC*H*_*2*_NCO), 3.58 (s, 2H, NCPhC*H*_*2*_N), 3.25–3.22 (m, 4H, CONC*H*_*2*_CH_2_N, NCOC*H*_*2*_N), 2.65 (t, 2H, *J* = 5.1 Hz, NC*H*_*2*_CH_2_NCO). ^13^C NMR (125 MHz, CDCl_3_, δ): 166.51, 138.49, 134.95, 133.49, 133.24, 132.30, 131.33, 129.58, 129.34, 128.86, 118.63, 112.69, 60.83, 57.19, 49.37, 48.90, 45.89. GC−MS (70 eV) (*m/z*) (rel. int.) 339 (M^+^, 11), 310 (12), 223 (42), 125 (100), 116 (91), 89 (32), 63 (5), 42 (42). HPLC Rt = 4.557 min (mobile phase: CH_3_CN/H_2_O 70/30; stationary phase: ZORBAX ECLIPSE Plus C18, analytical 4.6×250mm, 5 μm); rate = 1mL/min. The product was isolated by column chromatography (silica gel and EtOAc/hexane = 8.5/1.5 as the mobile phase).

#### 4-(3-Cyanobenzyl)-1-(4-(trifluoromethyl)benzyl)piperazin-2-one 22 (DA14).

Green pale oil; 31% yield. ^1^H NMR (500 MHz, CDCl_3_, δ): 7.64 (s, 1H, aromatic proton), 7.62–7.53 (m, 3H, aromatic protons), 7.49 (s, 1H, aromatic proton), 7.48–7.45 (m, 2H, aromatic protons), 7.44 (t, 1H, *J* = 7.65 Hz, aromatic proton), 4.65 (s, 2H, CF_3_PhC*H*_*2*_NCO), 3.61 (s, 2H, CNPhC*H*_*2*_N), 3.32–3.19 (m, 4H, NCOC*H*_*2*_N, CONC*H*_*2*_CH_2_N), 2.69 (t, 2H, *J* = 5.15 Hz, NC*H*_*2*_CH_2_NCO). ^13^C NMR (125 MHz, CDCl_3_, δ): 166.61, 137.47, 133.27, 132.32, 131.48, 131.47, 131.38, 130.04 (q, *J* = 32.1 Hz), 129.37, 129.31, 124.77 (q, *J* = 3.8 Hz), 124.55 (q, *J* = 3.7 Hz), 123.94 (q, *J* = 270.8 Hz), 118.59, 112.72, 60.80, 49.34, 49.18, 46.05. GC−MS (70 eV) (*m/z*) (rel. int.) 374 (4), 373 (M^+^, 17), 354 (5), 344 (31), 257 (100), 214 (8), 200 (6), 159 (82), 116 (99), 89 (22), 42 (42). HPLC Rt = 4.736 min (mobile phase: CH_3_CN/H_2_O 70/30; stationary phase: ZORBAX ECLIPSE Plus C18, analytical 4.6×250mm, 5 μm); rate = 1mL/min. The product was isolated by column chromatography (silica gel and EtOAc/hexane = 9/1 as the mobile phase).

#### 3-((4-(4-Fluorobenzyl)-3-oxopiperazin-1-yl)methyl)benzonitrile 23 (DA25).

Yellow oil; 35% yield. ^1^H NMR (500 MHz, CDCl_3_, δ): 7.62 (s, 1H, aromatic proton), 7.58–7.52 (m, 2H, aromatic protons), 7.43 (t, 1H, *J* = 7.7 Hz, aromatic proton), 7.25–7.20 (m, 2H, aromatic protons), 7.03–6.97 (m, 2H, aromatic protons), 4.55 (s, 2H, FPhC*H*_*2*_NCO), 3.57 (s, 2H, CNPhC*H*_*2*_N), 3.26–3.18 (m, 4H, NCOC*H*_*2*_N, CONC*H*_*2*_CH_2_N), 2.64 (t, 2H, *J* = 5.7 Hz, CONCH_2_C*H*_*2*_N). ^13^C NMR (125 MHz, CDCl_3_, δ): 166.65, 162.27 (d, *J* = 244.65 Hz), 138.62, 133.23, 132.27, 131.26, 129.90 (d, *J* = 8.05 Hz), 129.33, 129.32, 118.65, 115.56 (d, *J* = 21.35 Hz), 112.65, 60.82, 57.23, 49.36, 48.85, 45.87. GC−MS (70 eV) (*m/z*) (rel. int.) 323 (M^+^, 10), 294 (11), 207 (38), 116 (61), 109 (100), 89 (16), 42 (29). HPLC Rt = 3.823 min (mobile phase: CH_3_CN/H_2_O 70/30; stationary phase: ZORBAX ECLIPSE Plus C18, analytical 4.6×250mm, 5 μm); rate = 1mL/min. The product was isolated by column chromatography (silica gel and EtOAc/hexane = 9/1 as the mobile phase).

#### 4-(3-Cyanobenzyl)-1-(3,4-dichlorobenzyl)piperazin-2-one 24 (DA26)*.*

Orange oil; 98% yield. ^1^H NMR (300 MHz, CDCl3, δ): 7.66–7.62 (m, 1H, aromatic proton),7.61–7.53 (m, 2H, aromatic protons), 7.48–7.39 (m, 2H, aromatic protons),7.36 (d, 1H, *J* = 2.01 Hz, aromatic proton), 7.12 (dd, 1H, *J*_*1*_ = 8.22 Hz, *J*_*2*_ = 3.0 Hz, aromatic proton), 4.54 (s, 2H, bisClPhC*H*_*2*_NCO), 3.59 (s, 2H, CNPhC*H*_*2*_NCH_2_), 3.29–3.19 (m, 4H, NCOC*H*_*2*_N, CONC*H*_*2*_CH_2_N), 2.67 (t, 2H, *J* = 5.37 Hz, NC*H*_*2*_CH_2_NCO). ^13^C NMR (125 MHz, CDCl_3_, δ): 166.41, 137.18, 136.67, 133.33, 132.78, 132.39, 131.82, 131.45, 130.73, 130.06, 129.41, 127.56, 118.57, 112.75, 60.75, 56.99, 49.27, 48.63, 45.97. GC−MS (70 eV) (*m/z*) (rel. int.) 393 (M^+^, 11), 364 (24), 258 (14), 257 (100), 159 (89), 136 (64), 90 (46), 89 (22), 42 (53). HPLC Rt = 5.616 min (mobile phase: CH_3_CN/CH_3_COONH_4_ 70/30; stationary phase: ZORBAX ECLIPSE Plus C18, analytical 4.6×250mm, 5 μm); rate = 1mL/min. The product was isolated by column chromatography (silica gel and EtOAc/MeOH = 9.8/0.2 as the mobile phase).

#### 4-(3-Nitrobenzyl)-1-(3-(trifluoromethyl)benzyl)piperazin-2-one 25 (DA23).

Orange oil; 60% yield. ^1^H NMR (300 MHz, CDCl_3_, δ): 8.24–8.10 (m, 2H, aromatic protons), 7.67 (d, 1H, *J* = 7.6 Hz, aromatic proton), 7.59–7.44 (m, 5H, aromatic protons), 4.66 (s, 2H, CF_3_PhC*H*_*2*_NCO), 3.67 (s, 2H, NO_2_PhC*H*_*2*_N), 3.31–3.22 (m, 4H, NCOC*H*_*2*_N, CONC*H*_*2*_CH_2_N), 2.70 (t, 2H, *J* = 5.6 Hz, NC*H*_*2*_CH_2_NCO). ^13^C NMR (125 MHz, CDCl_3_, δ): 166.84, 148.40, 139.25, 137.54, 134.90, 131.48, 131.47, 130.96 (q, *J* = 32.1 Hz), 129.49, 129.31, 124.74 (q, *J* = 3.68 Hz), 124.51 (q, *J* = 3.7 Hz), 123.62, 123.97 (q, *J* = 271 Hz), 122.66, 60.79, 57.19, 49.41, 49.16, 46.18. GC−MS (70 eV) (*m/z*) (rel. int.) 393 (M^+^, 11), 364 (24), 258 (14), 257 (100), 159 (89), 136 (64), 90 (46), 89 (22), 42 (53). HPLC Rt = 5.385 min (mobile phase: CH_3_CN/H_2_O 70/30; stationary phase: ZORBAX ECLIPSE Plus C18, analytical 4.6×250mm, 5 μm); rate = 1mL/min. The product was isolated by column chromatography (silica gel and CH_2_Cl_2_/EtOAc = 9/1 as the mobile phase).

#### 4-(3-(Trifluoromethyl)benzyl)-1-(4-(trifluoromethyl)benzyl)piperazin-2-one 26 (DA29).

Orange oil; 25% yield. ^1^H NMR (300 MHz, CDCl_3_, δ): 7.65–7.42 (m, 6H, aromatic protons), 7.38 (d, 2H, *J* = 8.13 Hz, aromatic protons), 4.65 (s, 2H, CF_3_PhC*H*_*2*_NCO), 3.62 (s, 2H, CF_3_PhC*H*_*2*_N), 3.31–3.19 (m, 4H, NCOC*H*_*2*_N, CONC*H*_*2*_CH_2_N), 2.67 (t, 2H, *J* = 5.43 Hz, NC*H*_*2*_CH_2_NCO). ^13^C NMR (125 MHz, CDCl_3_, δ): 157.31, 140.33, 136.38, 132.62, 131.73, 131.02 (q, *J* = 32.63 Hz), 129.96 (q, *J* = 32.3 Hz), 129.62, 129.17, 128.59, 128.49, 128.32, 125.99, 125.97, 125.95, 125.92, 125.85, 125.83, 125.79, 125.68 (q, *J* = 3.7 Hz), 125.18, 125.15, 125.12, 124.98, 124.95, 124.92, 124.84, 124 (q, J = 270.56 Hz), 123.94 (q, J = 270.96 Hz), 61.00, 50.35, 49.22, 49.11, 49.73. GC−MS (70 eV) *(m/z*) (rel. int.) 416 (M^+^, 11), 387 (18), 257 (62), 200 (6), 159 (100), 109 (16), 42 (17). HPLC Rt = 4.777 min (mobile phase: CH_3_CN/H_2_O 70/30; stationary phase: ZORBAX ECLIPSE Plus C18, analytical 4.6×250mm, 5 μm); rate = 1mL/min. The product was isolated by column chromatography (silica gel and EtOAc/hexane = 7/3 as the mobile phase).

#### 1-(3-Bromo-4-fluorobenzyl)-4-(4-(trifluoromethyl)benzyl)piperazin-2-one 27 (DA42)*.*

Light brown solid, 42% yield. M.p. 83–86. ^1^H NMR (500 MHz, CD_3_OD, δ): 7.63 (d, 2H, *J* = 8.2 Hz, aromatic protons), 7.59–7.51 (m, 3H, aromatic protons), 7.14 (dd, 1H, *J*_*1*_ = 9.5 Hz, *J*_*2*_ = 1.7 Hz, aromatic proton), 7.03 (dd, 1H, *J*_*1*_ =8.2 Hz, *J*_*2*_ = 1.7 Hz, aromatic proton), 4.57 (s, 2H, Br,FPhC*H*_*2*_NCO), 3.68 (s, 2H, CF_3_PhC*H*_*2*_N), 3.34–3.31 (m, 2H, CONC*H*_*2*_CH_2_N), 3.23 (s, 2H, NCOC*H*_*2*_N), 2.72 (t, 2H, *J* = 5.6 Hz, NC*H*_*2*_CH_2_NCO). ^13^C NMR (125 MHz, CD_3_OD, δ): 167.91, 159.03 (d, *J* = 245.3 Hz), 141.51, 138.87 (d, *J* = 6.6 Hz), 129.38 ( q, *J* = 32 Hz), 128.33, 124.96 ( q, *J* = 3.7 Hz), 124.83 (d, *J* = 3.4 Hz), 124.26 (q, *J* = 269.7 Hz), 115.66 (q, *J* = 22.7 Hz), 107.18 (d, *J* = 20.9 Hz), 60.27, 56.41, 48.69, 46.09. GC−MS (70 eV) (*m/z*) (rel. int.) 446 (2), 445 (M^+^, 10), 444 (2), 443 (10), 418 (2), 417 (10), 416 (2), 415 (11), 288 (6), 287 (48), 286 (7), 285 (50), 189 (44), 188 (5), 187 (47), 160 (9), 159 (100), 109 (22), 108 (17), 107 (11), 42 (28). HPLC Rt = 4.893 min (mobile phase: CH_3_CN/H_2_O 70/30; stationary phase: ZORBAX ECLIPSE Plus C18, analytical 4.6×250mm, 5 μm); rate = 1mL/min. The product was isolated by column chromatography (silica gel and EtOAc/MeOH = 9.8/0.2 as the mobile phase).

#### General synthesis of 32–33.

A stirred suspension of 2,5-piperazinedione (0.876 mmol) and NaH (1.927 mmol) in anhydrous DMF (3 mL) was left at 0 °C for 30 minutes. After this time was added at room temperature the appropriate substituted benzyl bromide (1.199 mmol) and the reaction were stirring for 16h at room temperature. To the reaction mixture was added H_2_O and was concentrated under reduced pressure. EtOAc was added to the residue, and the solution was washed with a saturated solution of NH_4_Cl and with brine. The organic layer was dried on anhydrous Na_2_SO_4_, and the solvent was evaporated under reduced pressure. The resulting oil underwent column chromatography on silica gel to give 33 and 34.

#### 1,4-Dibenzylpiperazine-2,5-dione 33(DA28).

White solid, 58% yield. M.p. 176–180 °C. ^1^H NMR (500 MHz, CDCl_3_, δ): 7.37–7.29 (m, 6H, aromatic protons), 7.29–7.24 (m, 4H, aromatic protons), 4.58 (s, 4H, 2PhC*H*_*2*_NCO), 3.93 (s, 4H, 2NCOC*H*_*2*_NCO). ^13^C NMR (125 MHz, CDCl_3_, δ): 163.25, 134.92, 128.94, 128.54, 128.21, 49.29, 49.21. GC−MS (70 eV) (*m/z*) (rel. int.) 294 (M^+^, 19), 203 (1), 175 (4), 118 (6), 106 (6), 91 (100), 65 (11), 42 (5). HPLC Rt = 4.085 min (mobile phase: CH_3_CN/H_2_O 70/30; stationary phase: ZORBAX ECLIPSE Plus C18, analytical 4.6×250mm, 5 μm); rate = 1mL/min. The product was isolated by column chromatography (silica gel and EtOAc/hexane = 6/4 as the mobile phase).

#### 1,4-Bis(3-(trifluoromethyl)benzyl)piperazine-2,5-dione 34 (DA34).

White solid, 30% yield. M.p. 133–137 °C. ^1^H NMR (500 MHz, CDCl_3_, δ): 7.61–7.57 (m, 2H, aromatic protons), 7.53 (s, 2H, aromatic protons), 7.51–7.46 (m, 4H, aromatic protons), 4.64 (s, 4H, 2PhC*H*_*2*_NCO), 3.97 (s, 4H, 2CONC*H*_*2*_CON). ^13^C NMR (125 MHz, CDCl_3_, δ): 163.15, 135.96, 131.79, 131.42 (q, *J* = 32.3 Hz), 129.57, 125.20 (sextet, *J* = 3.7 Hz), 123.76 (q, *J* = 271 Hz), 49.32, 48.99. GC−MS (70 eV) (*m/z*) (rel. int.) 416 (M^+^, 11), 387 (18), 257 (62), 200 (6), 159 (100), 109 (16), 42 (17). HPLC Rt = 4.111min (mobile phase: CH_3_CN/H_2_O 70/30; stationary phase: ZORBAX ECLIPSE Plus C18, analytical 4.6×250mm, 5 μm); rate = 1mL/min. The product was isolated by column chromatography (silica gel and EtOAc/hexane = 6/4 as the mobile phase).

### Synthesis of 31 (DA45).

#### Tert-butyl 3-oxopiperazine-1-carboxylate 28 (DA41).

To a suspension of 2-oxopiperazine (0.200 g; 2 mmol) in anhydrous CH_2_Cl_2_ (5 mL), is added a solution of Di-*tert*-butyl dicarbonate (0.479 g; 2.2 mmol) in anhydrous CH_2_Cl_2_ (5 mL), dropwise at 0°C. The mixture was stirred for 3 h at room temperature. The organic layer was washed with brine. The organic extracts were dried over anhydrous Na_2_SO_4_, and the solvent evaporated under reduced pressure to give the compound **28**. White solid, 86% yield. M.p. 164–168 °C^37^. ^1^H NMR (300 MHz, CDCl_3_, δ): 6.41 (br s, 1H, N*H*CO), 4.10 (s, 2H, NHCOC*H*_*2*_N), 3.64 (t, 2H, *J* = 5 Hz, NCH_2_C*H*_*2*_NBoc), 3.35–3.44 (m, 2H, J = 5 Hz, NC*H*_*2*_CH_2_NBoc), 1.48 (s, 9H, C(C*H*_*3*_)_*3*_). HRMS (ESI) *m/z*: calc. for ESI-MS *m/z*: [C_9_H_16_N_2_O_3_+Na]^+^: 223.1081; ESI-MS-MS: 223.1054, 187.6279, 167.0421, 128.9449, 95.0572, 76.9985, 62.9815. ESI-MS m/z: [C_9_H_16_N_2_O_3_−H]^−^, 199.1083. ESI-MS-MS: 199.1082, 99.0566, 70.0300, 57.0345.

#### Tert-butyl 3-oxo-4-(4-(trifluoromethyl)benzyl)piperazine-1-carboxylate 29 (DA43)*.*

To a suspension of NaH, (60% dispersion in mineral oil, 0.048 g; 2 mmol) in anhydrous DMF (1 mL), was added a solution of **28** (0.200 g; 1 mmol) in anhydrous DMF (2.5 mL) at 0°C. The mixture was stirring for 30 min at the same temperature. Then, 4-(trifluoromethyl)benzyl bromide (0.286 g; 1.2 mmol) was added, and the mixture was left stirring at 100 °C, overnight. After cooling, to the reaction mixture was added H_2_O and was concentrated under reduced pressure. EtOAc was added to the residue, and the solution was washed with brine. The organic extracts were dried over anhydrous Na_2_SO_4_, and the solvent evaporated under reduced pressure. The product was isolated by column chromatography (silica gel and EtOAc/hexane = 6/4 as the mobile phase). Yellow solid, 53% yield. M.p.142–144 °C. ^1^H NMR (500 MHz, CDCl_3_, δ): 7.59 (d, 2H, *J* = 8.2 Hz, aromatic protons), 7.38 (d, 2H, *J* = 8.15 Hz, aromatic protons), 4.67 (s, 2H, CF_3_PhC*H*_*2*_N), 4.17 (s, 2H, NCOC*H*_*2*_N), 3.61 (t, 2H, *J* = 5.3 Hz, PhNC*H*_*2*_CH_2_N), 3.27 (t, 2H, *J* = 5.1 Hz, NC*H*_*2*_CH_2_NCO), 1.46 (s, 9H, C(C*H*_*3*_)_*3*_). HRMS (ESI) *m/z*: calc. for ESI-MS m/z: [C_17_H_21_F_3_N_2_O_3_+Na]^+^: 381.1406; ESI-MS-MS: 381.1405, 325.0776, 281.0870, 254.1047, 76.9973.

#### 1-(4-(Trifluoromethyl)benzyl)piperazin-2-one 30 (DA44).

An excess of CF_3_CO_2_H (2.81 mmol, 0.5 ml) was added to a solution of **29** in CH_2_Cl_2_ (3 mL). The reaction mixture was left stirring for 5h at room temperature. The solvent was concentrated under reduced pressure, and the product was used in the next step without any further purification. Yellow solid; quantitative yield. M.p. 126–130 °C. ^1^H NMR (500 MHz, CDCl_3_, δ): 7.59 (d, 2H, *J* = 7.9 Hz, aromatic protons), 7.36 (d, 2H, *J* = 7.9 Hz, aromatic protons), 4.66 (s, 2H, CF_3_PhC*H*_*2*_N), 3.92 (s, 2H, NCOC*H*_*2*_NH), 3.51 (t, 2H, *J* = 5.1 Hz, CONC*H*_*2*_CH_2_NH), 3.39 (t, 2H, *J* = 5.45Hz, HNCH_2_C*H*_*2*_N). GC−MS (70 eV) (*m/z*) (rel. int.) 258 (M^+^, 54), 188 (35), 159 (100), 109 (22), 99 (33), 56 (23), 42 (29).

#### 4-(2-(3,4-Dichlorophenyl)acetyl)-1-(4-(trifluoromethyl)benzyl)piperazin-2-one 31 (DA45).

To a solution of **29** (0.100 g; 0.39 mmol) in CH_2_Cl_2_ (3 mL) was added, dropwise, a solution of 3,4-dichlorophenylacetyl chloride in CH_2_Cl_2_ (2 mL) at 0 °C. The mixture was left stirring at room temperature, overnight. After this time, the solvent was concentrated under reduced pressure. The crude product was purified by fractional crystallization of hot/cold diethyl ether. The product **31** was obtained as a white solid, 53% yield. M.p.159–162 °C. ^1^H NMR (500 MHz, DMSO, δ): 7.74–7.66 (m, 2H, aromatic protons), 7.53 (t, 1H, *J* = 8.7 Hz, aromatic proton), 7.49–7.41 (m, 3H, aromatic protons), 7.19 (d, 2H, *J* = 8.25 Hz, aromatic protons), 4.62 (s, 2H, CF_3_PhC*H*_*2*_NCO), 4.28 (s, 1H, NCOC*H*HN), 4.11 (s, 1H, NCOCH*H*N), 3.80–3.74 (m, 3H, bisClC*H*_*2*_CO, CONC*H*HCH_2_NCH_2_), 3.68 (t, 1H, *J* = 4.9 Hz, CONCH*H*CH_2_NCH_2_), 3.31–3.28 (m, 1H, CONCH_2_C*H*HNCH_2_), 3.26 (t, 1H, *J* = 5 Hz, CONCH_2_CH*HCH*_*2*_). ^13^C NMR (125 MHz, DMSO, δ): 168.70, 165.70, 142.22, 137.27, 131.02, 131.97, 130.63, 130.49, 130.39, 129.53, 128.78, 128.71, 128.48, 128.23, 125.90, 125.88, 125.85, 125.78, 123.62, 46.33, 45.87, 42.66, 38.38, 38.22. HRMS (ESI) *m/z*: calc. for ESI-MS *m/z*: [C_20_H_17_Cl_2_F_3_N_2_O_2_+Na]^+^: 467.0526; ESI-MS-MS: 467.0524, 435.2174, 374,9542, 326.2443, 185.0144, 104.9924, 76.9978. ESI-MS *m/z*: [C_20_H_17_Cl_2_F_3_N_2_O_2_+Cl]^−^, 479.0302. ESI-MS-MS:479.0316, 443.0513, 283.0695, 257.0893, 184.9588, 158.9774, 82.0310. HPLC Rt = 4.509 min (mobile phase: CH_3_CN/H_2_O 70/30; stationary phase: ZORBAX ECLIPSE Plus C18, analytical 4.6×250mm, 5 μm); rate = 1mL/min.

#### General synthesis of 36–37.

To a stirred suspension of 1-acetylpiperazine (2.34 mmol) and K_2_CO_3_ (3.04 mmol) in CH_3_CN (14 mL) was added the appropriate benzyl bromide (2.57 mmol). The reaction mixture was stirred at room temperature for 3 h. The reaction progress was monitored by TLC (CH_2_Cl_2_/MeOH = 9.5/0.5 as mobile phase). Then, the resulting solid was filtered and repeatedly washed with EtOAc. The organic filtrate was concentrated under reduced pression, and the resulting oil underwent to chromatography on silica gel and CH_2_Cl_2_/MeOH = 9.5/05 as mobile phase.

#### 1-(4-(4-Chlorobenzyl)piperazin-1-yl)ethan-1-one 36 (DA5).

Orange oil; 57% yield. ^1^H NMR (300 MHz, CDCl_3_, δ): 7.34–7.20 (m, 4H, aromatic protons), 3.62 (t, 2H, *J* = 5. Hz, CONC*H*_*2*_), 3.49 (s, 2H, ClPhC*H*_*2*_N), 3.45 (t, 2H, *J* = 5.2 Hz, CONC*H*_*2*_), 2.52–2.31 (m, 4H, C*H*_*2*_NC*H*_*2*_), 2.07 (s, 3H, C*H*_*3*_CO). GC−MS (70 eV) (*m/z*) (rel. int.) 252 (M^+^, 7), 180 (33), 168 (26), 125 (100), 85 (30), 56 (13), 42 (19).

#### 3-((4-Acetylpiperazin-1-yl)methyl)benzonitrile 37 (DA6).

Orange oil; 55% yield. ^1^H NMR (300 MHz, CDCl_3_, δ): 7.66 (m, 1H, aromatic proton), 7.61–7.51 (m, 2H, aromatic protons), 7.43 (t, 1H, *J* = 7.7 Hz, aromatic proton), 3.63 (t, 2H, *J* = 5 Hz, CONC*H*_*2*_), 3.54 (s, 2H, CNPhC*H*_*2*_N), 3.47 (t, 2H, J = 5.1 Hz, CONC*H*_*2*_), 2.52–2.30 (m, 4H, C*H*_*2*_NC*H*_*2*_), 2.08 (s, 3H, C*H*_*3*_CO). GC−MS (70 eV) (*m/z*) (rel. int.) 243 (M^+^, 6), 200 (10), 171 (81), 159 (39), 116 (100), 89 (22), 56 (23), 42 (27).

### BIOLOGICAL SECTION

#### Purification of *h*ClpP.

*h*ClpP was expressed with an N-terminal 2x(His6-thrombin)-SUMO tag in BL21(DE3) pRIL cells. 1L cultures were inoculated at a concentration of 1:100 in LB supplemented with 30 μg/mL of Kanamycin and grown at 37°C with shaking until the logarithmic growth phase. Protein expression was induced with 1 mM IPTG and maintained at 18 °C for 16–18 hours with shaking at 135 rpm. Cells were harvested by centrifugation and resuspended in buffer A (25 mM Tris, pH 7.4, 0.5 M NaCl, 10 % (v/v) glycerol) supplemented with 0.05 g of lysozyme (BioShop), a SIGMAFAST protease inhibitor cocktail tablet (SigmaAdlrich) and 50 μL of DNaseI (Roche). The homogenized culture was lysed on ice with three cycles of sonication (Branson sonifier 450; 3 minutes per cycle, 50 % duty). All subsequent purification steps were performed at 4 °C unless otherwise stated. The lysate was centrifuged for 30 min to remove debris. The supernatant containing SUMO-tagged *h*ClpP was incubated with Ni-Sepharose beads (Cytiva) for 15 min and purified using buffer A mixed with a standard imidazole gradient. The eluted protein was dialyzed in 4 L for 18 hours in buffer B (25 mM Tris, pH 7.4, 10% glycerol, 0.1 M NaCl, 1 mM EDTA, 1 mM DTT). ULP1 was added to the dialysis (1:50 mass ratio with eluted protein) to cleave the 2x(His_6_-thrombin)-SUMO tag. The cleaved tag was subsequently removed by rebinding to Ni-Sepharose beads. The purified, untagged *h*ClpP was concentrated and stored at −80°C until use.

#### *h*ClpP + DA29 crystallization and structural refinement.

*h*ClpP was incubated at 10 mg/mL in a stoichiometric ratio of 1:3 with **26** (DA29) dissolved in DMSO. This solution was then mixed 1:1 with crystallization solution (0.2 M MgCl_2_ , 25%w/v PEG 3350, 0.1 M BIS-TRIS pH 5.5, on plates for sitting drop vapour diffusion crystallization at room temperature. The resulting crystals were frozen in liquid nitrogen using the crystallization solution supplemented with an additional 20% glycerol as cryo-protectant. Diffraction data was collected with 0.979338 Å wavelength X-rays at the National Synchrotron Light Source II at Brookhaven National Laboratory (Upton, New York, USA) Beamline 17-ID-2. Diffraction data was indexed, integrated, and scaled using autoPROC 1.0.5^38^ Molecular replacement was performed in Phenix Phaser^39^. The search model was a structure of *h*ClpP bound to ONC201 (PDB ID 6DL7). The ONC201 molecules were removed from the PDB prior to molecular replacement. Subsequent model building and refinements were performed in Coot^40^ and Phenix^41^ and involved the optimization of atomic coordinates, B-factor, and occupancy. Clear difference density for DA29 was found in the electron density maps and was confirmed by calculating a composite omit map. The final structure has good geometry, with 96% of residues in Ramachandran favoured regions and no Ramachandran outliers. Data collection and refinement statistics are summarized in Table S2.

#### *h*ClpP activity test.

FITC-casein assay was performed to assess the potency of the proteolytic activity of *h*ClpP. In a black, flatbottom, 96-well plate, purified 2 μM *h*ClpP in assay buffer (AB) (50 mM *N*-(2-hydroxyethyl)piperazine-*N*′-ethanesulfonic acid (HEPES), pH 7.5, 300mMKCl, 1mMDTT, 15% v/v glycerol; AB) preincubated at 37 °C for 15 min was added to 5 μL of the tested compounds in dimethyl sulfoxide (DMSO) at different concentrations (1–100 μM)and incubated for 15 min at 37 °C under shaking. As a control, three wells were filled with 5 μL of DMSO. The kinetic measurement was started after adding 2 μM fluorogenic FITC-casein (Merck, C3777), used as the enzyme substrate. The FITC fluorescence from the hydrolyzed FITC-casein was recorded over 60 min at 37 °C on a TecanInfinite M200 Pro (λ_ex_ = 485 nm, λ_em_ = 535 nm, gain: 60). The slope in the linear range between 480 and 1200 s was determined and plotted against time. The EC_50_ for each tested compound was calculated using GraphPad Prism 7.05. Results are reported as the mean of two independent experiments performed in triplicate.

#### Cellular Thermal Shift Assay.

Caco-2 cells were preincubated for 1 h with compounds solubilized in DMSO at a final concentration of 20 μM or DMSO alone as control experiment at the concentration of 0.04%. 100 μL of treated cells was aliquoted into polymerase chain reaction (PCR) tubes (Qiagen) and heated in a SensoQuest 96-well Thermal Cycler (Diatech Pharmacogenetics) at the indicated temperature range for 3 min. Immediately following heating, the aliquots were equilibrated to room temperature (3 min). Subsequently, the cells were rapidly frozen in liquid nitrogen to extract their contents and immediately centrifuged at 14,000 rpm for 20 min at 4 °C to separate soluble from insoluble fragments. The supernatant was carefully aspirated and subjected to Western blotting under reducing conditions. The soluble fractions were solubilized in 2× Laemmli and separated onto 10% Tris-Glycine-SDS minigels (Bio-Rad) and then transferred onto 0.2 μm nitrocellulose membranes using the Trans-Blot Turbo Transfer System (Bio-Rad). Membranes were blocked for 1 h at room temperature with blocking buffer (5% non-fat dry milk, 0.1% Tween-20 in Tris-buffered saline, TBS). The membranes were probed with anti-hClpP polyclonal primary antibody (Product #PA5–52722, Thermo Fisher) at a dilution of 1:1000 at 4 °C overnight, diluted in 5% bovine serum albumin in TBST. After incubation time, membranes were washed ×3 with TBST and incubated with a secondary peroxidase antibody (1:3000 antirabbit) for 1 h at room temperature. After repeated washing, the membranes were treated with the Clarity Western ECL substrate (Bio-Rad) according to the manufacturers instructions, and the blot was visualized by iBright FL1000 Imaging Systems (Thermo Fisher Scientific).

#### Cell Cultures.

Patient-derived DIPG cell cultures (SU-DIPG-36, SU-DIPG-50) were kindly provided by Prof. Michelle Monje of Institutional Review Board (Stanford University and Prof. Javad Nazarian). The cells were cultured as a monolayer in media that was changed once a week at 37 °C in 5% CO_2_ by using Tumor Stem Media composed by a 1:1 ratio of DMEM/F12 (Invitrogen)/Neurobasal (-A) (Invitrogen), B27 (-A) (LifeTechnologies, Milan, Italy), 20 ng/mL human basic fibroblast growth factor (Life Technologies), 20 ng/mL recombinant human epidermal growth factor (Life Technologies), 10 ng/mL platelet-derived growth factor-AA, 10 ng/mL platelet-derived growth factor-BB (Life Technologies), and 20 ng/mL heparin (StemCell Technologies, Milan, Italy).19 Caco-2 cells were grown in Dulbeccos high-glucose modified Eagle medium, composed of 10% fetal bovine serum, 2 mM glutamine, 100 U/mL penicillin, and 0.1 mg/mL streptomycin (all components purchased from Euroclone, Milan, Italy).

#### Cytotoxic Assay.

The cytotoxicity assay was conducted seeding cells at a density of 10,000 cells/well for 24 h in a 96-well plate (Corning, NY, USA) and incubated overnight at 37 °C in a 5% CO_2_ atmosphere. Subsequently, the culture medium was replaced with 100 μL of fresh medium containing dilutions of drug ranging from 1 to 100 μM and incubated at 37 °C for 72 h. Drug-solvent DMSO was added to each control to evaluate a possible solvent cytotoxicity. After the incubation time with each compound, CCK-8 (10 μL) was added to each well, and after 3–4 h of incubation at 37 °C, the absorbance values at λ = 450 nm were determined on the Tecan Infinite 200 microplate reader Tecan Infinite 200. IC_50_ values were obtained by using GraphPad Prism software.

#### Drug Transport Experiments.

The experiment started with the preparation of the Caco-2 monolayer, which occurred by seeding the cells (20,000/well) in Millicell plates (Millipore, Milan, Italy). Its growth was followed for 21 days by changing the medium occasionally and measuring its transepithelial electrical resistance daily using an epithelial voltohmmeter (Millicell-ERS) until at least 1000W was reached. After 21 days, the plate was washed twice with Hanks balanced salt solution (HBSS) (Invitrogen). After the second wash, the wells were filled with buffer, and the plate was kept at 37 °C for 30 min. After the incubation time, the HBSS buffer was replaced with the solutions of the compounds to be tested at a concentration equal to 10^−4^ M. The plates were placed in an incubator at 37 °C for 2 h. The apparent permeability (Papp) was calculated in units of nm/s. The calcein-AM assay^42^ revealed that **26** (DA29) shows only a weak interaction with P-gp (EC_50_ = 71 μM), indicating that it is poorly retained in cells^43,44^. Its limited effect on P-gp efflux suggests minimal transporter-mediated restriction, consistent with its low intracellular accumulation.

#### Dynamic Permeability Assay Using Caco-2 Cells in the LiveBox2 Bioreactor System.

LiveFlow^®^ peristaltic pump was purchased from IVTech Srl (IVTech Srl., Massarosa, LU, Italy) together with the LiveBox2 (LB2) bioreactor. The LiveBox2 is composed of three parts: an apical chamber, with an inlet, an outlet and a wet volume of 1 mL; a basal chamber, with an inlet and an outlet tube and a wet volume of 1.5 mL and a membrane holder, which is placed between the two chambers. The two chambers are made in polydimethylsiloxane (PDMS; Sylgard 184, Dow Corning, Silverstar, Italy), a bio- compatible silicone polymer, and they set up two independent fluidic circuits, respectively. Each circuit is connected to a pump and a mixing chamber, which serves as a media reservoir and for oxygenation. The holder is composed of two annuli, which snugly holds a polyethylene terephthalate (PET) semi-permeable membrane (pore size: 0.45 μm; 2 × 10^6^ pores/cm^2^; ipCELLCULTURE, it4ip, Louvain-la-Neuve, Belgium) and it is placed between the two chambers.

After all the components were sterilized and the LiveBox2 was assembled under a laminar flow hood, the membrane was treated with a 0.01% solution of Poly-L-Lysine (Sciencell Research Laboratories 0413, Catalog No.NC9951372) for 1h at 37 °C. Then, the membrane was washed two times with sterile water.

Caco-2 cells were seeded with a density of 5×10^4^ cells/cm^2^ onto the membrane in the LB2. The media was changed every three days, and the cells were used for experiments after 8 days from seeding. To precondition the cell monolayer to dynamic conditions, the LB2 was connected to the peristaltic pump LiveFlow, using a laminar flow of 100 μL/min and the entire system was placed in the cell culture incubator at 37 °C with 5% CO_2_. After 24 hours, both apical and basal circuits were emptied out and media in the mixing chambers was rapidly replaced with fresh media for the basal circuit and with media containing compound for the apical circuit. The flow rate was increased to 150 μL/min, which according to the manufacturer should provide an average shear stress of around 6 × 10^−4^ Pa on the membrane, which is within the physiological range.

When the circuits are filled, media samples of 100 μL were withdrawn from upper circuit at various time points (0, 1, 2, 4, 6 h) and analyzed using HPLC performed on an Agilent 1260 Infinity instrument equipped with a 1260 DAD VL + detector.

The analysis was conducted in isocratic conditions by using acetonitrile H_2_O = 50:50 (THX6) as a mobile phase at a constant flow rate of 1 mL/min (injection volume: 50 μL). The stationary phase was constituted by a Zorbax Eclipse Plus C18 column, 250 × 4.6 mm Agilent^®^ (Santa Clara, CA, USA). UV detection was made at λ = 230 nm.

#### ROS production in SU-DIPG-36 cells.

Flow cytometry analysis was performed to determine the intracellular production of ROS as reported^45^. Briefly, SU-DIPG-36 cells were seeded at a density of 500.000 cells per dish; after 24 hours, cells were treated with the investigated compounds at the selected concentrations. After the above mentioned timepoints SU-DIPG-36 cells were washed with warm DPBS and subsequently incubated with DCFH-DA 1μM for 60 minutes at 37 °C and 5% CO_2_, protected from light. The cells were then washed with warm buffer, detached and centrifuged as described above, before being resuspended for assay run in the flow cytometer.

#### Immunoblotting analyses.

Whole-cell extracts were obtained by solubilization in lysis buffer [50 mM Tris-HCl, pH 7.4; 150 mM NaCl; 1 mM EDTA; 1% Triton X-100; cOmplete^™^ Protease Inhibitor Cocktail (Roche)], followed by incubation at 4 °C for 30 min. Extracts were cleared by 15 min centrifugation at 12,000× g. Whole cell proteins (20–40 μg) were fractionated onto 12% Tris-Glycine-SDS minigels. Immunodetection was carried out by following primary antibodies: anti-ClpX (Abcam), anti-ClpP (Abcam), anti-total OXPHOS cocktail (Abcam), anti-TFAM (Cell Signaling Technology), anti-mS29 (Life Technology), anti-uL13 (ProteinTech), anti-VDAC1 (ProteinTech), and anti-β-actin (Sigma-Aldrich). Chemiluminescent detection was performed using Clarity Western ECL substrate (Bio-Rad), and signals were revealed by the ChemiDoc MP Imaging System (Bio-Rad).

#### Relative quantitation of mtDNA level.

Mitochondrial DNA relative quantification was performed as described in Bruni et al.^46^. Melting curve analysis was performed to ensure the amplification specificity; the relative quantification was carried out according to the Pfaffl mathematical model^47^.

#### Mitoribosomal subunits and ClpXP complex assembly analysis.

Isokinetic sucrose gradient analyses were performed as described in Loguercio Polosa et al.^48^. Briefly, whole-cell extracts (1 mg) were loaded onto a 10–30% (v/v) sucrose gradient and subjected to centrifugation in an Optima L-100K ultracentrifuge (Beckman Coulter) using the SW 40Ti rotor at 100,000× g for 2 h and 15 min at 4 °C. For each experiment, eleven fractions were collected and analyzed by immunoblotting.

#### Establishment of PDT-O.

Fresh biopsy samples used for PDT-O development were obtained from Hospices Civils de Lyon Biobank (CRB-HCL, BB-0033–00046) in the context of patient diagnosis, with all necessary regulatory approvals and informed consents for all patients. PDT-O models were established and cultured as previously described^23^. Briefly, minced tissues were digested at 37 °C with gentle vortex for 30–90 min. PDT-O were then established in 96-well plates using appropriate culture medium^19^. Medium was changed twice a week and PDT-O were split every 1 to 2 weeks when reaching a diameter of 800–1000 μm

#### Chemosensitivity profiling of PDT-O.

For dose-response experiments, PDT-O were dissociated and seeded at 4000 cells/well in 96-well black ULA plates (Corning, cat. no. 4515). After 4 days, fully formed PDT-O were treated with serial dilutions of **26** (DA29) and ONC201 for 72 h. Cell viability was then measured using the CellTiter-Glo^®^ 3D Cell Viability Assay (Promega, cat. no. G9683), in accordance with the manufacturer’s instructions, and normalized with untreated (vehicle) negative control wells as 100% viability. Luminescence acquisitions were performed on a Spark^®^ microplate reader (Tecan) with a 400-ms exposition and auto-attenuation. Data were obtained from two independent experiments performed in three technical replicates per condition. Dose-response curves were obtained using Prism 8.0.2 (GraphPad).

#### Radioligand binding experiments *h*D2R and *h*D3R.

HEK293 cells stably expressing human D_2L_R or D_3_R were grown in a 50:50 mix of DMEM and Ham’s F12 culture media, supplemented with 20 mM HEPES, 2 mM L-glutamine, 0.1 mM non-essential amino acids, 1X antibiotic/antimycotic, 10% heat-inactivated fetal bovine serum, and 200 μg/mL hygromycin (Life Technologies, Grand Island, NY) and kept in an incubator at 37 °C and 5% CO_2_. Upon reaching 80−90% confluence, cells were harvested using premixed Earle’s balanced salt solution with 5 mM EDTA (Life Technologies) and centrifuged at 3,000 rpm for 10 min at 21°C. The supernatant was removed, and the pellet was resuspended in 10 mL hypotonic lysis buffer (5 mM MgCl_2_, 5 mM Tris, pH 7.4 at 4 °C) and centrifuged at 18,000 rpm (∼21,000g) for 20 min at 4 °C. The pellet was then resuspended in binding buffer. Bradford protein assay (Bio-Rad, Hercules, CA) was used to determine the protein concentration. Membranes were diluted to 500 μg/mL, in fresh EBSS binding buffer made from 8.7 g/L Earle’s Balanced Salts without phenol red (US Biological, Salem, MA), 2.2 g/L sodium bicarbonate, pH to 7.4, and stored in a −80 °C freezer for later use. On the test day, each test compound was diluted into half-log serial dilutions using the 30% dimethyl sulfoxide (DMSO) vehicle. Membranes were diluted in fresh binding buffer (100–200 μg/mL) and radioligand competition experiments were conducted in 96-well plates containing 300 μL fresh binding buffer, 50 μL of the diluted test compound, 100 μL of membranes (10−20 μg/well total protein for both hD_2L_R and hD_3_R), and 50 μL of radioligand diluted in binding buffer ([^3^H]-*N*-methylspiperone: 0.4 nM final concentration for all the hD_2_-like receptor subtypes; Novandi Chemistry AB, Södertälje, Sweden). Aliquots of radioligands solution were also quantified accurately in each experiment replicate, to determine how much radioactivity was added, taking in account the experimentally determined counter efficiency. Nonspecific binding was determined using 10 μM (+)-butaclamol (Sigma-Aldrich, St. Louis, MO), and total binding was determined with the 30% DMSO vehicle (3% final concentration). All compound dilutions were tested in triplicate, and the reaction incubated for 60 min at RT. The reaction was terminated by filtration through PerkinElmer Uni-Filter-96 GF/C, presoaked for the incubation time in 0.5% polyethylenimine (PEI), using a Revvity Unifilter-96 Cell Harvester (Revvity, Waltham, MA, USA). The filters were washed thrice with 3 mL (3 times ~1 mL/well) of ice-cold binding buffer. Revvity MicroScint-20 Scintillation Cocktail (65 μL) was added to each well, and filters were counted using a PerkinElmer MicroBeta Microplate Counter (Revvity, Waltham, MA, USA). IC_50_ values for each compound were determined from dose−response curves, and *K*_i_ values were calculated using the Cheng−Prusoff equation. *K*_d_ values were determined via separate [^3^H]-NMSP saturation binding experiments (*K*_d_ for D_2_R = 0.320 ± 0.0618 nM; *K*_d_ for D3R = 0.262 ± 0.0296 nM). When a complete inhibition could not be achieved at the highest tested concentrations, *K*_i_ values have been extrapolated by constraining the bottom of the dose−response curves (=0% residual specific binding) in the nonlinear regression analysis. These analyses were performed using GraphPad Prism 10.5 for Macintosh (GraphPad Software, San Diego, CA, USA). All results were rounded to the third significant figure. *K*_i_ values were determined from at least three independent experiments and are reported as the mean ± standard error of the mean (SEM). Compounds reported as “not active” displayed <50% inhibition, at the highest tested concentration of 10 μM, in a single experiment performed in triplicate.

#### Computational Studies.

Computational studies were performed using FLAP (v2.2.2) in structure-based virtual screening mode. Molecular Interaction Fields (MIFs) were calculated with the GRID algorithm on the crystal structure of *h*ClpP in complex with **ZK53** (PDB code: 8HGK, 1.90 Å). Docking validation was conducted with AutoDock Vina (via UCSF Chimera) using grid boxes defined from the FLAP-identified pocket and visualized with PyMOL^49–51^.

#### Lipidomic analyses.

Total lipids of the cell lines untreated and treated with ONC201 and 26 (DA29) were extracted by using the protocol reported in our previous research studies^2^. The chloroform extract containing lipid substances was analyzed by using GC and HPLC analytical techniques for an exhaustive characterization expressed in terms of fatty acids and intact lipids. For GC analyses, lipid extracts were derivatized and analyzed by GC-MS and GC-FID as reported in Miciaccia et al.^2^. With respect to HPLC-MS/MS investigation, the lipid extract was reconstituted by using a volume of 500 μL of isopropanol and sonicated for 10 min. The analysis was performed on a Shimadzu Ultra High-Performance Liquid Chromatograph Nexera X-2 system (Shimadzu), including two LC-30 AD dual-plunger parallel-flow pumps, a DGU-20A5R degasser, a CTO-20AC column oven and a SIL-30AC auto-sampler. The UHPLC system was coupled to a LCMS-8060 triple quadrupole mass spectrometer equipped with ESI interface (Shimadzu). Mobile phases were: (A) 20 mM ammonium formate and (B) 2-propanol/acetonitrile/20 mM ammonium formate (60:36:4 *v/v/v*) with 0.1% formic acid. The gradient program was: 0–6 min, 80–100% B (held for 16 min). The flow rate was 0.4 mL min^−1^; the column used was an Ascentis Express C18, 100 × 2.1 mm, 2.7 μm dp (Merck Life Science, Darmstadt, Germany), the oven was set at 40 °C and the injection volume was 5 μL. MS and MS/MS acquisitions were performed using ESI source in positive (+) ionization mode, with the following parameters: interface temperature, 450 °C; CDL temperature, 250 °C; heat block temperature, 200 °C; nebulizing gas flow (N2), 3 L min^−1^; drying gas flow (N2), 5 L min^−1^; acquisition range, 350–1250 *m/z*.

Additional MS/MS experiments were optimized through the injection of single PL standards, as previously reported^52^. Data acquisition and processing was handled by the LabSolution ver. 5.95 software (Shimadzu Europa, Duisburg, Germany). The LIPID MAPS Structure Database (LMSD) was employed for compound identification (available at https://www.lipidmaps.org/data/structure/) by searching the following ion type: [M+H]^+^ and [M+Na]^+^ for LPC/PC/SM, and [M+NH4]^+^ for TG. Data scedasticity was evaluated by using a two tailed F test (P values < 0.05 were considered as heteroscedastic). Statistically significant differences were evaluated by using a two tailed t test (P values < 0.05 were considered statistically significant). Statistical analysis was performed by using Excel 365 (Microsoft).

## Supplementary Material

Supplementary Files

This is a list of supplementary files associated with this preprint. Click to download.

• SupplementaryInformation27November.docx

## Figures and Tables

**Table 1.** EC_50_ (μM) and percentage activation values (%) of *h*ClpP of the dibenzylpiperazine targets, determined by a fluorimetric cell-free assay based on the cleavage of FITC-labeled casein substrate.

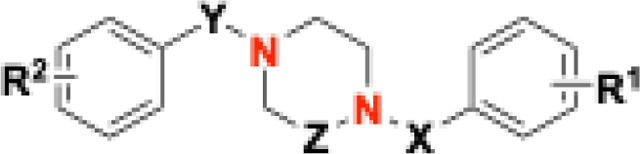						
Compd	Z	X	Y	R^2^	R^1^	*h*ClpP EC_50_ (μM)^a^ (% activation at 100μM)
**1** (DA1)	CH_2_	CH_2_	CH_2_	4-Cl	4-Cl	>100 (9)
**2** (DA4)	CH_2_	CH_2_	CH_2_	3-CN	3-CN	>100 (19)
**4** (DA7)	CH_2_	CH_2_	CH_2_	3-CF_3_	3-CN	>100 (27)
**7** (DA12)	CH_2_	CO	CO	H	H	>100 (0)
**8** (DA15)	CH_2_	CO	CO	3-CN	3-CN	>100 (11)
**9** (DA17)	CH_2_	CO	CO	4-Cl	3-CN	>100 (15)
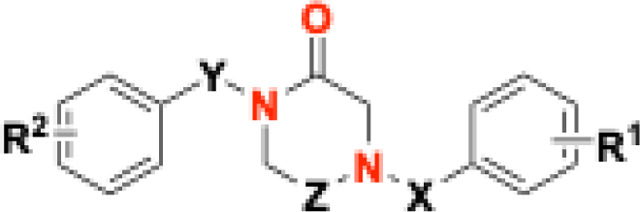						
Compd	Z	X	Y	R2	R1	*h*ClpP EC_50_ (μM)^a^ (% activation at 100μM)
**10** (DA9)	CH_2_	CH_2_	CH_2_	3-CN	3-CN	>100 (28)
**11** (DA24)	CH_2_	CH_2_	CH_2_	3-CF_3_	3-CF_3_	12 ± 5.15 (85)
**12** (DA32)	CH_2_	CH_2_	CH_2_	2-F	2-F	>100 (15)
**13** (DA36)	CH_2_	CH_2_	CH_2_	4-F	4-F	>100 (43)
**14** (DA33)	CH_2_	CH_2_	CH_2_	2-Br	2-Br	>100 (24)
**15** (DA35)	CH_2_	CH_2_	CH_2_	4-Br	4-Br	6.08 ± 0.51 (91)
**16** (DA53)	CH_2_	CH_2_	CH_2_	4-Cl	4-Cl	>100 (14)
**21** (DA11)	CH_2_	CH_2_	CH_2_	4-Cl	3-CN	15 ± 0.68 (63)
**22** (DA14)	CH_2_	CH_2_	CH_2_	3-CF_3_	3-CN	30 ± 0.15 (58)
**23** (DA25)	CH_2_	CH_2_	CH_2_	4-F	3-CN	22 ± 9.01 (43)
**24** (DA26)	CH_2_	CH_2_	CH_2_	3,4-diCl	3-CN	14 ± 2.81 (57)
**25** (DA23)	CH_2_	CH_2_	CH_2_	3-CF_3_	4-NO_2_	20 ± 1.22 (65)
**26** (DA29)	CH_2_	CH_2_	CH_2_	4-CF3	3-CF_3_	0.85 ± 0.14 (95)
**27** (DA42)	CH_2_	CH_2_	CH_2_	4-CF3	3-Br, 4-F	>100 (18)
**31** (DA45)	CH_2_	COCH2	CH_2_	4-CF3	3,4-diCl	>100 (24)
**33** (DA28)	CO	CH_2_	CH_2_	H	H	>100 (28)
**34** (DA34)	CO	CH_2_	CH_2_	3-CF_3_	3-CF_3_	>100 (13)

a EC_50_ values are presented as means ± SEM from at least 3 independent experiments, each performed in triplicate.

**Table 2.** Paediatric brain tumour-derived cell line IC_50_ after single drug treatment for a 72-hour incubation with piperazines, assessed with CCK-8 cell viability assay.^a^

Compd	SU-DIPG-36 IC_50_ μM ± SEM (% inhibition at 100 μM)	SU-DIPG-50 IC_50_ μM ± SEM (% inhibition at 100 μM)
**11** (DA24)	55 ± 6.25 (82.1)	15 ± 3.06 (68.1)
**15** (DA35)	64.9 ± 5.28 (91)	46.8 ± 1.63 (73.5)
**21** (DA11)	>100 (36)	72 ± 2.87 (64)
**22** (DA14)	25 ± 2.51 (51)	75 ± 3.82 (53)
**23** (DA25)	>100 (51)	>100 (22)
**24** (DA26)	7.8 ± 0.56 (80)	41 ± 1.43 (57)
**25** (DA23)	61 ± 2.93 (60)	>100 (53)
**26** (DA29)	4.0 ± 1.20 (86)	11.6 ± 0.12 (69.1)
**33** (DA28)	>100 (35)	>100 (26)
**ONC201**	37 ± 2.5 (82)	25 ± 12.3 (66)

a Data are expressed as the mean of three independent experiments and SEM values are reported.
